# Supplementary material for: Contact Cluster Modeling of Allosteric Communication in PDZ Domains
Source: J Phys Chem B. 2026 Jan 10;130(4):1121–30. doi: 10.1021/acs.jpcb.5c07737 (PMC12862798; doi:10.1021/acs.jpcb.5c07737)
Supplement: Supplementary file 1 [file jp5c07737_si_001.pdf]

Supporting Information: Contact cluster modeling of allosteric communication in PDZ domains

Emanuel Dorbath, Fabian Rudolf, Adnan Gulzar, and Gerhard Stock*

Biomolecular Dynamics, Institute of Physics, University of Freiburg, 79104 Freiburg, Germany

E-mail: stock@physik.uni-freiburg.de

Contents

1 Primary and secondary structures **S-2**

2 PDZ3 **S-3**

3 PDZ3WT **S-10**

4 PDZ3L6 **S-14**

5 PDZ2S **S-19**

6 PDZ2L **S-25**

7 Diffusive dynamics **S-36**

References **S-37**

1 Primary and secondary structures

Primary structure of all considered systems. Residues marked in blue represent β-sheets, in red α-helices, and in green the ligand. The anchor residues of the photoswitch are cysteines denoted by 'C'.

PDZ3:
GLGEEDIPREPRRIVIHRGSTGLGFNIVGGENGEGIFISFILAGGPADLSGELRK
GDQILSVNGVDLRNASHEQAAIALKNAGQTVTIIAQYKPCEYSRFECK — KETWV

PDZ3WT:
The same as PDZ3, but with the photoswitch anchor residues are not mutated to Cys, i.e., they remain as Glu95 and Ala102.

PDZ3L6:
Same as PDZ3 but with ligand KKETWV.

PDZ2L:
PKPGDIFEVELAKNDNSLGISVTGGVNTSVRHGGIYVKAVIPQGAAESDGRIHKGD
RVLAVNGVSLEGATHKQAVETLRNTGQVVHLLLEKGQS — RWAKSEAKECEQVSCV

PDZ2S:
GPKPGDIFEVELAKNDNSLGICVTGGVNTSVRHGGIYVKAVIPQGAAESDGRIHKGD
RVLAVNGVSLEGATHKQAVCTLRNTGQVVHLLLEKGQSPT

Table S1: Secondary structures of PDZ3, PDZ2S, and PDZ2L. Note, for example, that the $\beta_2\beta_3$-loop has different lengths in PDZ2 and PDZ3.

**Structure**	β_1	β_2	β_3	α_1	β_4	β_5	α_2	β_6	α_3
**PDZ3**	12–17	25–28	36–40	46–50	56–62	65–66	72–81	85–92	94–99
**PDZ2S**	7–13	21–24	36–41	46–50	58–62	65–66	74–81	85–91	
**PDZ2L**	6–12	20–24	35–40	45–49	57–61	64–65	71–80	84–90	

2 PDZ3

MD simulations

Details of equilibrium and nonequilibrium MD simulations of PDZ3 are given in Refs. S1,S2. Here we selected 100 statistically independent starting configurations from $8 \times 10\,\mu$s long *cis* equilibrium simulations for which at $t = 0$ the transition is initiated utilizing the potential energy surface switching method.[S3] Following the deactivation of the applied potential energy after only 0.5 ps, we produced $100 \times 1\,\mu$s-long trajectories, from which 10 are extended to $10\,\mu$s. For better convergence at long times, $12 \times 10\,\mu$s-long nonequilibrium simulations were added in in Ref. S2. Using a write-out time step of $\Delta t = 20$ ps, we obtain in total $0.45 \cdot 10^7$ for the $90 \times 1\,\mu$s-long trajectories and $1.1 \cdot 10^7$ data points for the $22 \times 10\,\mu$s-long trajectories.

MoSAIC clustering

The contact distances of interest were selected based on the $100 \times 1\,\mu$s long trajectories with the criterion being that for 10 % of the total simulation time the contact distance is below 0.45 nm, excluding the two nearest neighbors ($|i - j| > 2$, for residues i and j). This yields 403 unique contact distances, for which we performed the correlation-based feature selection MoSAIC,[S4] using the constant Potts model for the objective function, see Methods in the main text. We used as similarity measure the Pearson correlation, and only every 5th frame of the MD data was taken. The latter has little to no effect on the clustering as MoSAIC works very well with sparse data, but significantly improves the computational time required. Using a resolution parameter $\gamma = 0.5$ and requiring that a cluster contains at least 4 distances, we obtained 8 localized and highly correlated contact cluster (Fig. S1). Since cluster C8 is dominated by residues of the highly flexible N-terminus that is not of functional relevance, only cluster C1 to C7 were considered in the further analysis, the contact distances of which are listed in Tab. S2.

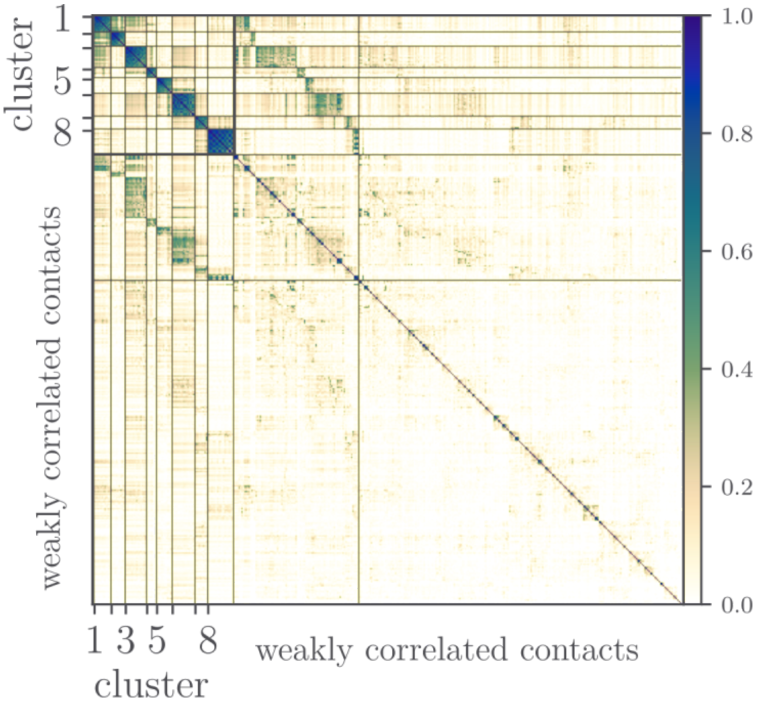

Figure S1: PDZ3 – MoSAIC analysis, using 403 contact distances and a resolution parameter of $\gamma = 0.5$. The top left corner shows the desired MoSAIC clusters, while the other contact distances are either weakly (middle) or hardly (bottom right) correlated.

Table S2: PDZ3 – List of contact distances included in the main MoSAIC clusters.

Cluster	Contacts
C1	$r_{94,102}$, $r_{97,102}$, $r_{28,101}$, $r_{28,102}$, $r_{39,102}$, $r_{55,102}$, $r_{101,(-3)}$, $r_{101,(-4)}$, $r_{102,(-3)}$, $r_{103,(-3)}$, $r_{103,(-4)}$
C2	$r_{28,100}$, $r_{29,100}$, $r_{30,100}$, $r_{31,100}$, $r_{34,100}$, $r_{35,100}$, $r_{37,100}$, $r_{96,100}$, $r_{97,100}$
C3	$r_{29,71}$, $r_{33,68}$, $r_{33,69}$, $r_{33,70}$, $r_{33,71}$, $r_{34,68}$, $r_{34,69}$, $r_{35,67}$, $r_{35,68}$, $r_{35,69}$, $r_{35,70}$, $r_{36,79}$, $r_{58,68}$, $r_{59,68}$
C4	$r_{27,(-3)}$, $r_{27,(-4)}$, $r_{28,(-3)}$, $r_{28,(-4)}$, $r_{29,(-4)}$, $r_{72,(-4)}$
C5	$r_{81,86}$, $r_{82,86}$, $r_{83,86}$, $r_{18,81}$, $r_{77,81}$, $r_{62,82}$, $r_{63,82}$, $r_{78,82}$, $r_{79,82}$, $r_{79,83}$
C6	$r_{18,82}$, $r_{18,83}$, $r_{18,86}$, $r_{21,24}$, $r_{21,25}$, $r_{21,42}$, $r_{21,43}$, $r_{21,45}$, $r_{21,46}$, $r_{18,(0)}$, $r_{18,(-1)}$, $r_{20,(-1)}$, $r_{21,(-1)}$, $r_{22,(-1)}$, $r_{24,(0)}$
C7	$r_{47,51}$, $r_{47,52}$, $r_{14,50}$, $r_{50,53}$, $r_{51,54}$, $r_{16,52}$, $r_{15,52}$, $r_{25,53}$
C8	$r_{1,95}$, $r_{2,95}$, $r_{1,98}$, $r_{2,51}$, $r_{1,51}$, $r_{1,102}$, $r_{2,98}$, $r_{2,52}$, $r_{3,54}$, $r_{2,102}$, $r_{1,100}$, $r_{1,48}$, $r_{3,95}$, $r_{2,94}$, $r_{2,53}$, $r_{2,54}$, $r_{2,97}$

C1

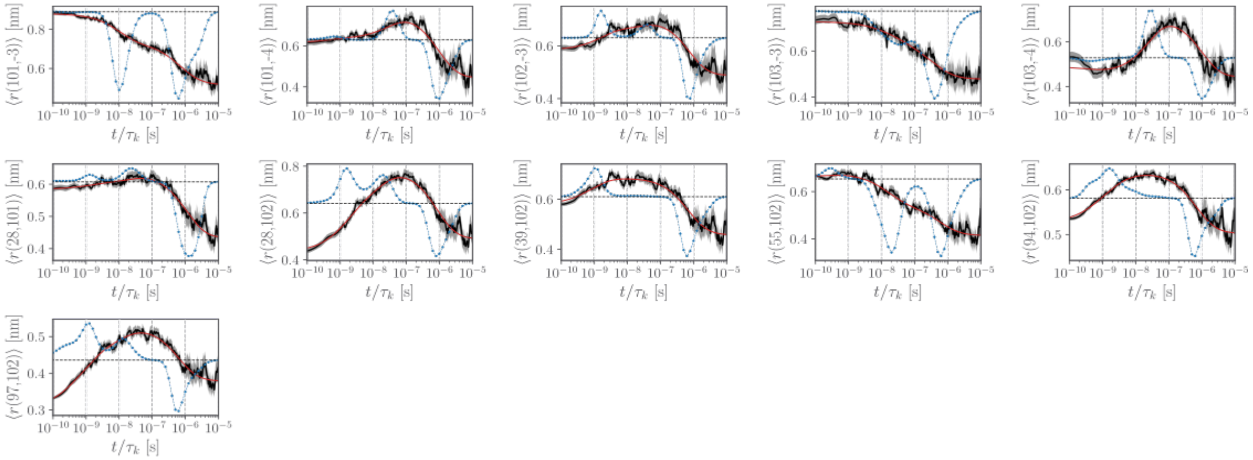

C2

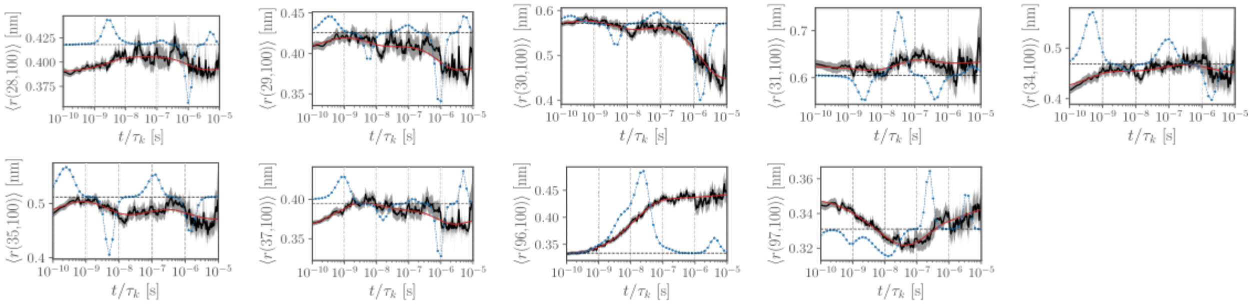

C3

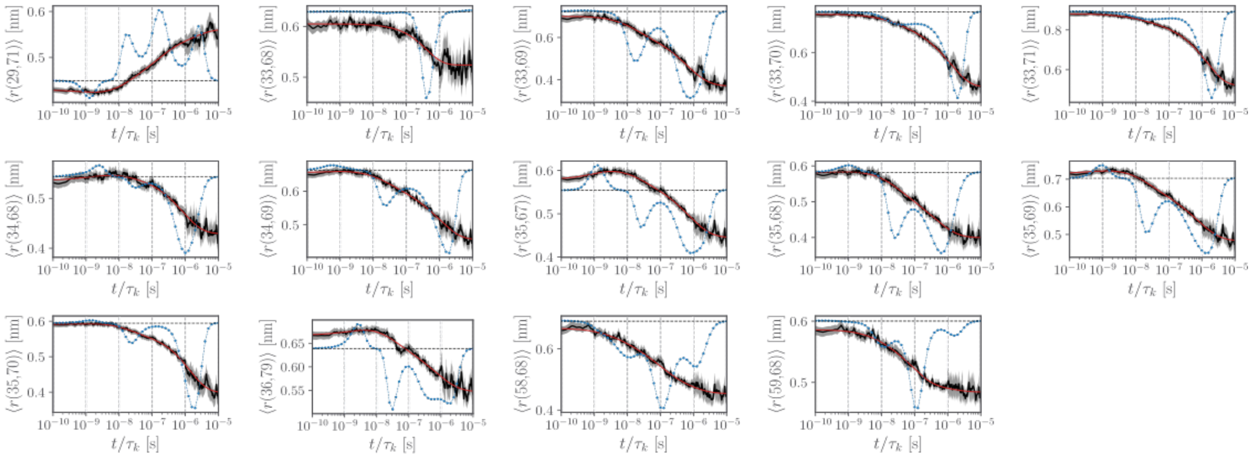

C4

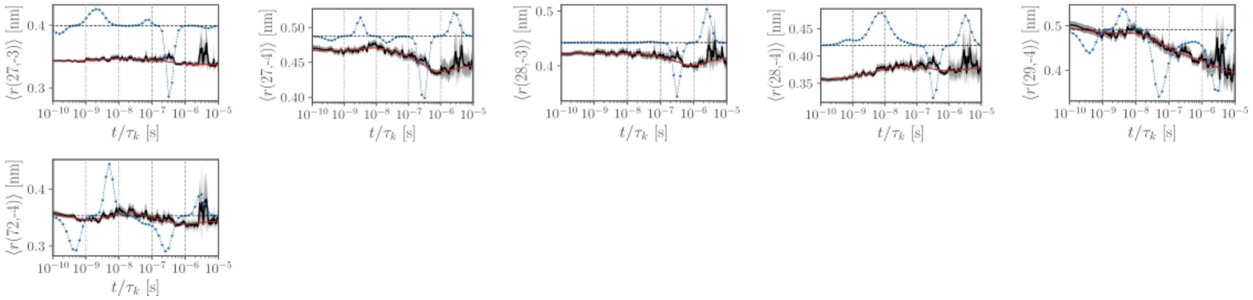

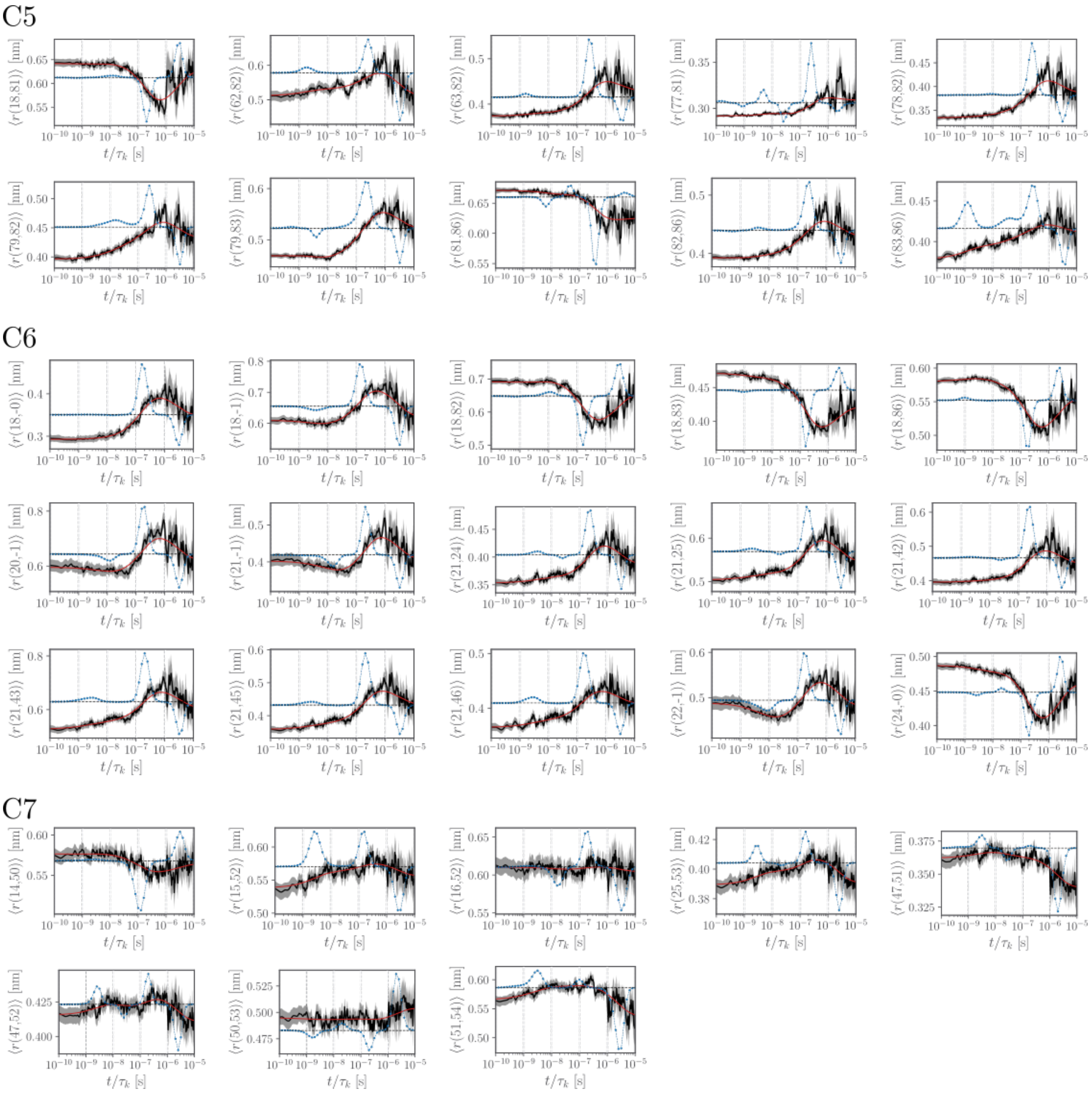

Figure S2: PDZ3 – Time evolution of contact distances in clusters C1 to C7, along with a timescale analysis using the regularization parameter $\lambda = 50$.

Inverse contact distances

We performed the above analysis of PDZ3 also for inverse contact distances $\langle r \rangle \to 1/\langle r \rangle$. The regularization parameter was adjusted to $\lambda = 10$. This is supposed to be more sensitive to smaller changes,[S5] however, we did not find significant differences between timescales obtained from contact distances or from inverse ones.

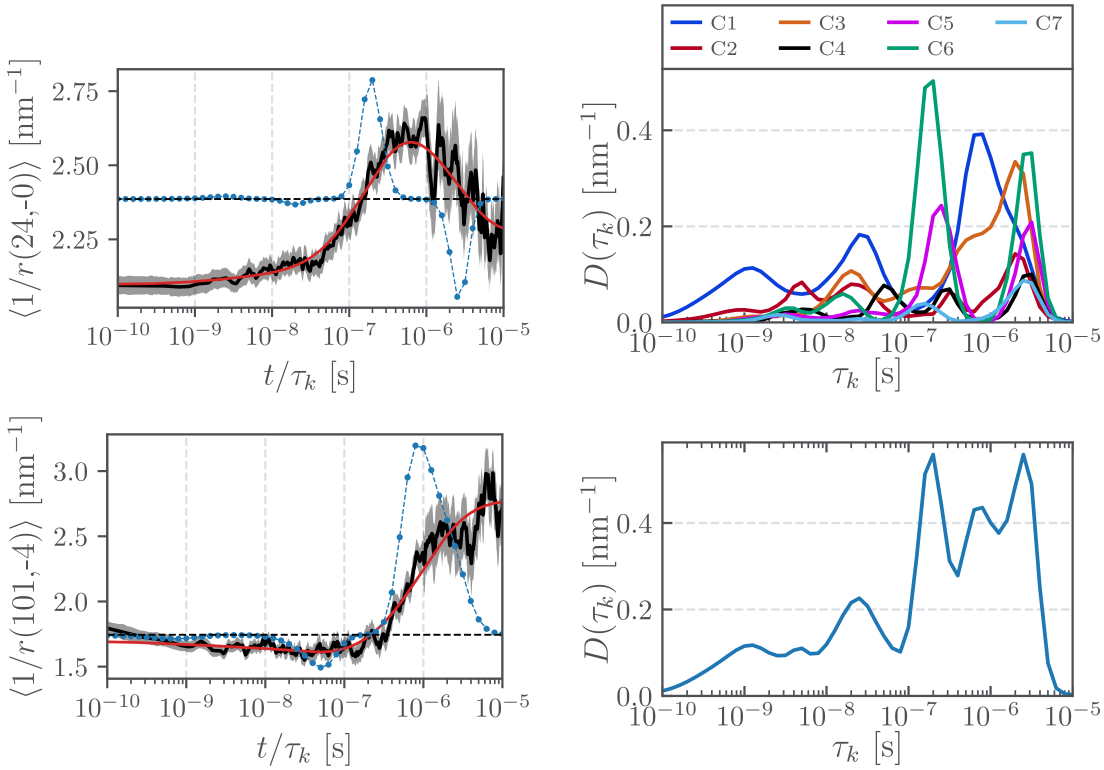

Figure S3: PDZ3 – (Left) Time evolution and timescale analysis of two selected inverse distances $1/\langle r \rangle$ of PDZ3 with $\lambda = 10$. (Right) The obtained timescales of the individual distances as well as the resulting dynamical content are matching closely the ones shown in Fig. 1 in the main text.

MoSAIC analysis of equilibrium simulations

Using the same 403 contact distances as identified from the nonequilibrium simulations, we also performed a MoSAIC clustering using the *cis* and *trans* equilibrium data of PDZ3,[S1] which contain in total 8×10^6 frames covering a total of 160 μs. For the MoSAIC analysis, the Pearson correlation is used with every 5th frame and a resolution parameter of $\gamma = 0.4$. Apart from the seven main clusters also identified by the nonequilibrium data (Fig. S1), we found several smaller clusters (only 5 distances each) whose dynamics are dominated by two neighboring anchor residues, as well as two large clusters involving the highly fluctuating N-terminus.

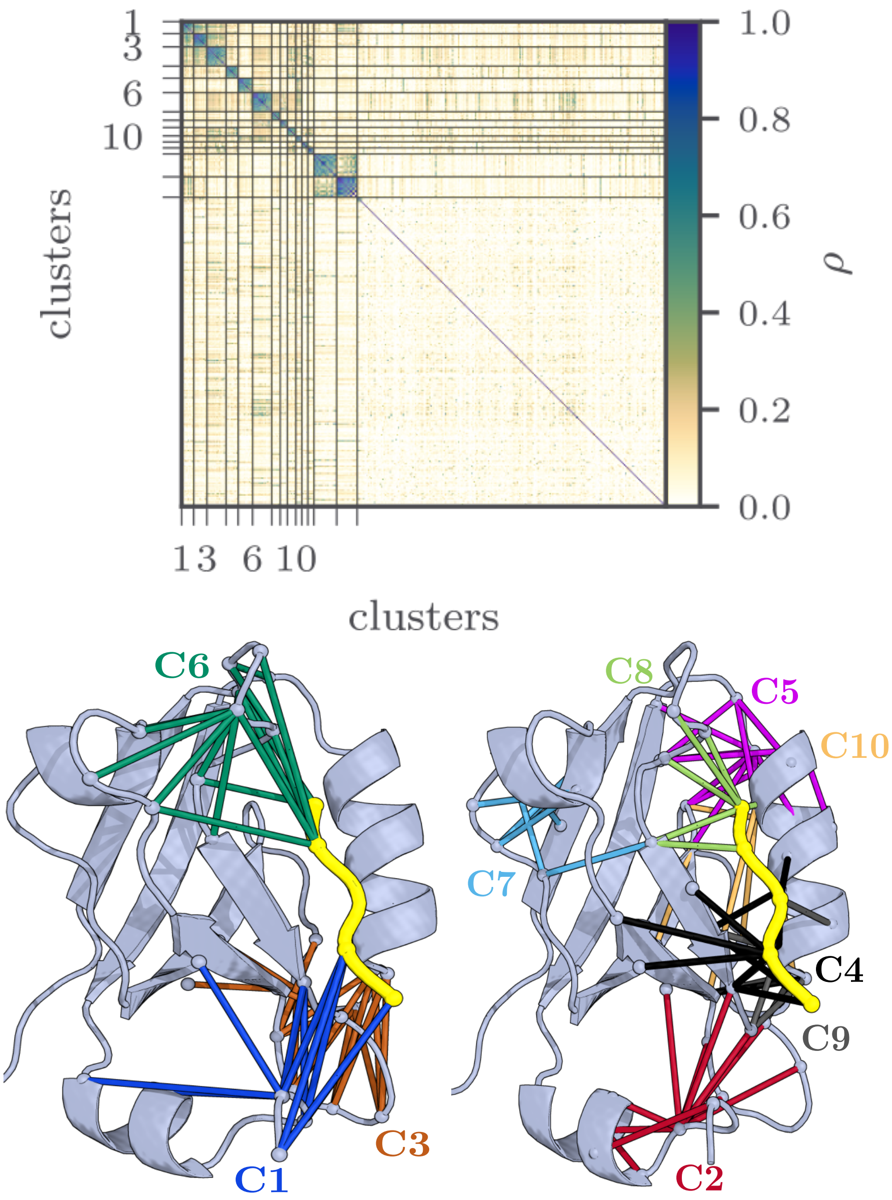

Figure S4: PDZ3 EQ – MoSAIC clustering with $\gamma = 0.4$. The respective distances for each cluster are indicated in the bottom.

Table S3: PDZ3 EQ – Contact clusters with $\gamma = 0.4$.

**Cluster**	**Contacts**
C1	$r_{28,101}$, $r_{28,102}$, $r_{39,102}$, $r_{94,102}$, $r_{97,102}$, $r_{101,-4}$, $r_{101,-3}$, $r_{102,-3}$, $r_{103,-4}$, $r_{103,-3}$
C2	$r_{28,100}$, $r_{29,100}$, $r_{30,100}$, $r_{31,100}$, $r_{34,100}$, $r_{35,100}$, $r_{37,100}$, $r_{96,99}$, $r_{96,100}$, $r_{97,100}$, $r_{98,102}$
C3	$r_{30,36}$, $r_{32,69}$, $r_{32,70}$, $r_{32,71}$, $r_{33,69}$, $r_{33,70}$, $r_{33,71}$, $r_{34,68}$, $r_{34,69}$, $r_{34,70}$, $r_{35,67}$, $r_{35,68}$, $r_{35,69}$, $r_{35,70}$, $r_{58,68}$, $r_{59,68}$
C4	$r_{26,-3}$, $r_{27,-4}$, $r_{27,75}$, $r_{28,-4}$, $r_{28,-3}$, $r_{36,71}$, $r_{39,-3}$, $r_{40,-3}$, $r_{72,-2}$, $r_{76,-2}$
C5	$r_{62,82}$, $r_{63,81}$, $r_{63,82}$, $r_{77,81}$, $r_{78,82}$, $r_{79,82}$, $r_{79,83}$, $r_{80,83}$, $r_{81,86}$, $r_{82,85}$, $r_{82,86}$, $r_{83,86}$
C6	$r_{18,-1}$, $r_{18,0}$, $r_{18,21}$, $r_{19,22}$, $r_{19,84}$, $r_{20,-1}$, $r_{21,-1}$, $r_{21,24}$, $r_{21,25}$, $r_{21,42}$, $r_{21,43}$, $r_{21,45}$, $r_{21,46}$, $r_{22,-1}$, $r_{24,0}$, $r_{42,-1}$
C7	$r_{14,50}$, $r_{15,52}$, $r_{16,52}$, $r_{25,53}$, $r_{47,51}$, $r_{47,52}$, $r_{50,53}$
C8	$r_{21,0}$, $r_{22,0}$, $r_{23,0}$, $r_{25,-1}$, $r_{25,0}$, $r_{79,0}$
C9	$r_{27,72}$, $r_{28,72}$, $r_{29,71}$, $r_{29,72}$, $r_{30,70}$, $r_{36,70}$, $r_{73,-2}$
C10	$r_{36,79}$, $r_{59,62}$, $r_{63,78}$, $r_{67,79}$, $r_{70,75}$
C11	$r_{30,71}$, $r_{30,72}$, $r_{31,71}$, $r_{31,72}$, $r_{31,73}$
C12	$r_{28,35}$, $r_{29,34}$, $r_{29,35}$, $r_{30,34}$, $r_{34,58}$
C13	$r_{2,55}$, $r_{3,54}$, $r_{3,55}$, $r_{3,92}$, $r_{4,54}$, $r_{4,55}$, $r_{4,92}$, $r_{5,54}$, $r_{5,92}$, $r_{5,94}$, $r_{6,94}$, $r_{6,95}$, $r_{7,92}$, $r_{7,94}$, $r_{8,92}$, $r_{8,93}$, $r_{8,94}$, $r_{8,95}$, $r_{9,54}$
C14	$r_{1,48}$, $r_{1,51}$, $r_{1,95}$, $r_{1,98}$, $r_{1,100}$, $r_{1,102}$, $r_{2,51}$, $r_{2,52}$, $r_{2,53}$, $r_{2,54}$, $r_{2,94}$, $r_{2,95}$, $r_{2,97}$, $r_{2,98}$, $r_{2,102}$, $r_{3,95}$, $r_{3,97}$
C15	$r_{39,97}$, $r_{93,97}$, $r_{94,98}$, $r_{95,99}$

3 PDZ3WT

MD simulations

We performed $4 \times 1\,\mu$s-long equilibrium simulations of PDZ3 in its wild-type (WT) form, i.e., without a photoswitch attached at the α_3-helix.[S1] The effective time step was 20 ps yielding a total of $2 \cdot 10^5$ MD frames.

MoSAIC clustering

We used the same 403 contact distances as for PDZ3 to facilitate easy comparison. The MoSAIC analysis with a resolution parameter of $\gamma = 0.35$ yields similar clusters as for PDZ3, despite the equilibrium nature of the trajectories. Of the 10 largest clusters, two are dominated by the long and flexible N-terminus which is of little interest.

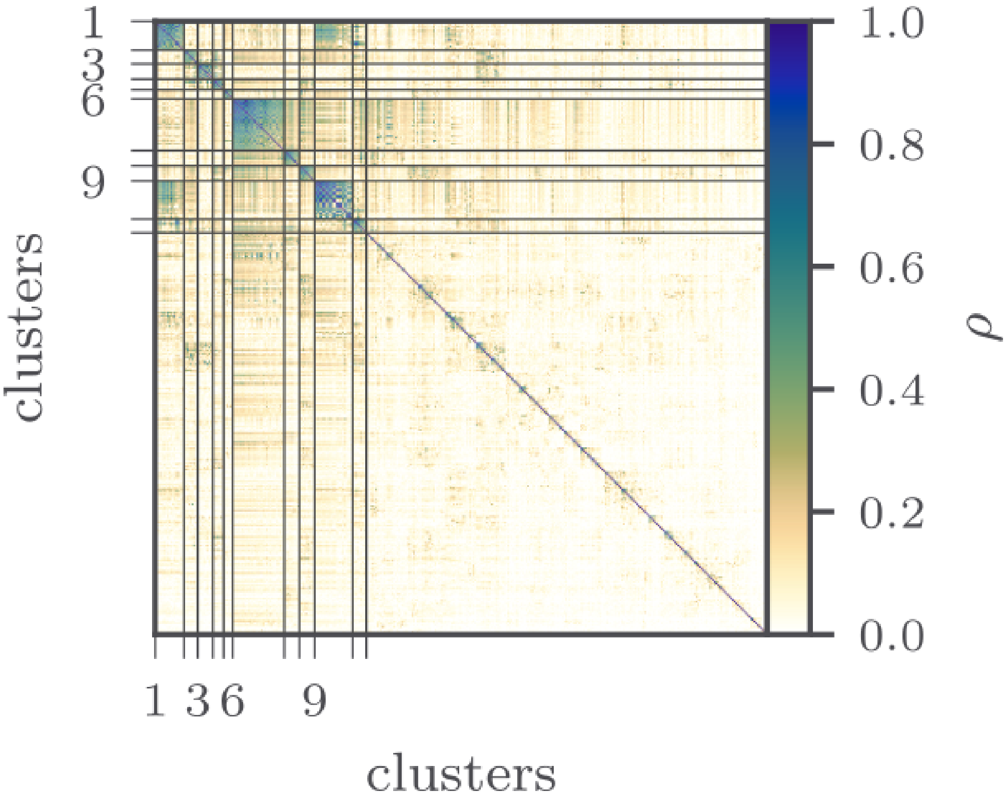

Figure S5: PDZ3WT – MoSAIC analysis of 403 contact distances with a resolution parameter of $\gamma = 0.35$.

Table S4: PDZ3WT – Contact cluster distances derived for $\gamma = 0.35$.

Cluster	Contacts
C1	$r_{5,54}$, $r_{7,92}$, $r_{7,94}$, $r_{8,92}$, $r_{8,93}$, $r_{8,94}$, $r_{8,95}$, $r_{9,92}$, $r_{28,102}$, $r_{39,102}$, $r_{94,102}$, $r_{95,102}$, $r_{97,101}$, $r_{98,102}$, $r_{99,102}$, $r_{101,-4}$, $r_{102,-3}$, $r_{103,-4}$, $r_{103,-3}$
C2	$r_{28,35}$, $r_{29,34}$, $r_{29,35}$, $r_{30,34}$, $r_{30,35}$, $r_{34,58}$, $r_{34,100}$, $r_{35,67}$, $r_{35,100}$
C3	$r_{30,33}$, $r_{32,69}$, $r_{32,70}$, $r_{32,71}$, $r_{33,68}$, $r_{33,69}$, $r_{33,70}$, $r_{33,71}$, $r_{34,68}$, $r_{34,69}$
C4	$r_{35,68}$, $r_{35,69}$, $r_{35,70}$, $r_{58,68}$, $r_{59,68}$, $r_{60,68}$, $r_{61,68}$
C5	$r_{63,82}$, $r_{77,81}$, $r_{78,82}$, $r_{79,82}$, $r_{81,86}$, $r_{82,86}$
C6	$r_{17,22}$, $r_{18,-1}$, $r_{18,0}$, $r_{18,21}$, $r_{18,81}$, $r_{18,82}$, $r_{18,83}$, $r_{18,86}$, $r_{19,22}$, $r_{19,46}$, $r_{19,84}$, $r_{20,-1}$, $r_{21,-1}$, $r_{21,24}$, $r_{21,25}$, $r_{21,42}$, $r_{21,43}$, $r_{21,45}$, $r_{21,46}$, $r_{22,-1}$, $r_{23,45}$, $r_{24,-1}$, $r_{24,0}$, $r_{24,45}$, $r_{24,46}$, $r_{25,0}$, $r_{25,42}$, $r_{25,47}$, $r_{25,79}$, $r_{26,42}$, $r_{27,0}$, $r_{42,-1}$, $r_{79,83}$, $r_{80,83}$
C7	$r_{12,91}$, $r_{14,50}$, $r_{15,52}$, $r_{16,52}$, $r_{25,53}$, $r_{47,50}$, $r_{47,51}$, $r_{47,52}$, $r_{50,53}$, $r_{52,57}$
C8	$r_{36,75}$, $r_{36,79}$, $r_{59,62}$, $r_{59,79}$, $r_{62,82}$, $r_{62,86}$, $r_{63,78}$, $r_{67,78}$, $r_{67,79}$, $r_{70,75}$
C9	$r_{1,48}$, $r_{1,51}$, $r_{1,95}$, $r_{1,98}$, $r_{1,100}$, $r_{1,102}$, $r_{2,51}$, $r_{2,52}$, $r_{2,53}$, $r_{2,54}$, $r_{2,55}$, $r_{2,94}$, $r_{2,95}$, $r_{2,97}$, $r_{2,98}$, $r_{2,102}$, $r_{3,54}$, $r_{3,55}$, $r_{3,92}$, $r_{3,95}$, $r_{3,97}$, $r_{4,54}$, $r_{4,55}$, $r_{4,92}$, $r_{5,95}$
C10	$r_{5,9}$, $r_{5,92}$, $r_{5,94}$, $r_{6,94}$, $r_{6,95}$, $r_{9,54}$, $r_{12,54}$, $r_{31,34}$, $r_{51,54}$

MoSAIC clustering of local C_α-distances

"Local" C_α-distances are below 0.8 nm for at least 10% of the simulation time. Using a resolution parameter of $\gamma = 0.5$, the MoSAIC analysis yields very similar clusters as obtained for contact distances above.

Table S5: PDZ3WT – Cluster obtained for C_α-distances below 0.8 nm with $\gamma = 0.5$.

Cluster	Contacts
C1	$r_{28,102}$, $r_{28,103}$, $r_{29,102}$, $r_{29,103}$, $r_{97,101}$, $r_{98,101}$, $r_{98,102}$, $r_{98,103}$, $r_{99,102}$, $r_{99,103}$, $r_{102,-3}$
C2	$r_{28,100}$, $r_{34,100}$, $r_{39,101}$, $r_{95,99}$, $r_{96,99}$, $r_{96,100}$, $r_{97,100}$
C3	$r_{35,69}$, $r_{35,70}$, $r_{36,68}$, $r_{36,70}$, $r_{59,67}$, $r_{59,68}$, $r_{60,67}$, $r_{60,68}$, $r_{61,67}$, $r_{61,68}$
C3'	$r_{29,34}$, $r_{29,35}$, $r_{34,68}$, $r_{35,59}$, $r_{35,60}$, $r_{35,67}$, $r_{35,68}$
C4	$r_{27,-3}$, $r_{28,-4}$, $r_{28,-3}$, $r_{29,-4}$, $r_{29,-3}$
C4'	$r_{30,35}$, $r_{30,36}$, $r_{30,37}$, $r_{31,36}$
C5	$r_{62,75}$, $r_{62,78}$, $r_{63,78}$, $r_{63,79}$, $r_{65,78}$
C5'	$r_{62,82}$, $r_{78,82}$, $r_{79,82}$, $r_{79,83}$, $r_{83,86}$
C6	$r_{16,23}$, $r_{17,23}$, $r_{18,21}$, $r_{18,23}$, $r_{18,24}$, $r_{19,22}$, $r_{19,23}$, $r_{19,24}$, $r_{19,45}$, $r_{19,46}$, $r_{19,83}$, $r_{19,84}$, $r_{19,85}$, $r_{20,45}$, $r_{20,84}$, $r_{21,24}$, $r_{21,25}$, $r_{21,42}$, $r_{21,45}$, $r_{22,-1}$, $r_{22,25}$, $r_{23,0}$, $r_{24,-1}$, $r_{24,0}$, $r_{24,46}$, $r_{25,0}$, $r_{80,84}$
C7	$r_{16,50}$, $r_{47,51}$, $r_{47,52}$, $r_{48,51}$, $r_{50,53}$, $r_{51,54}$

Using all C_α-distances and normal mode analysis

Using all $N(N-1)/2$ C_α distances in the MoSAIC analysis amounts to about 5000 coordinates, most of which are of minor importance. Hence we ranked all distances by their variance and included only the first ~ 1000 distances that contain 80 % of the total variance. Performing a MoSAIC analysis with $\gamma = 0.7$ on this data, we obtain 12 clusters with at least 20 contact distances. (We increased the threshold for noise clusters as we have about $\times 2.5$ as many input features.) Representative clusters are shown in Fig. S6 where most C_α distances span away from one (or a few) anchor residues to many residues at the opposite end of the protein. Thus, these numerous and redundant distances primarily reflect the overall protein motion.

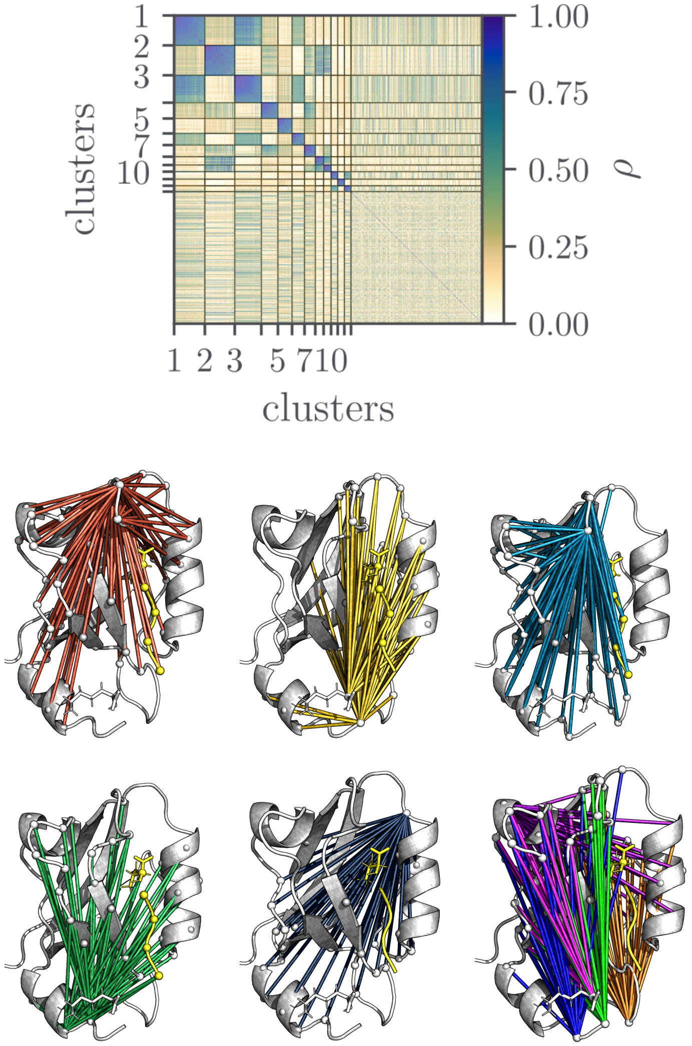

Figure S6: PDZ3WT – (Top) MoSAIC analysis of 996 variance-ranked C_α-distances, resulting in a cumulative relative variance of 80 %. (Bottom) The obtained contact clusters. The top row shows clusters C1, C2 and C3, the bottom row clusters C4, C5 and C6-C10.

Alternatively, protein dynamics can be described by the lowest eigenvibrations obtained from a normal mode (NM) analysis.[S6–S8] Adopting a C_α-atom representation of the equilibrium structure, we calculated the normal modes and display the 20 C_α-distances with the highest variance of the mode in Fig. S7. Similar to the all-C_α MoSAIC clusters shown above, we again observe anchor residues from which the normal mode originates. While the individual clusters and modes are not identical, we find a high similarity between C1 and NM1, C2 and NM8 as well as C4 and NM4.

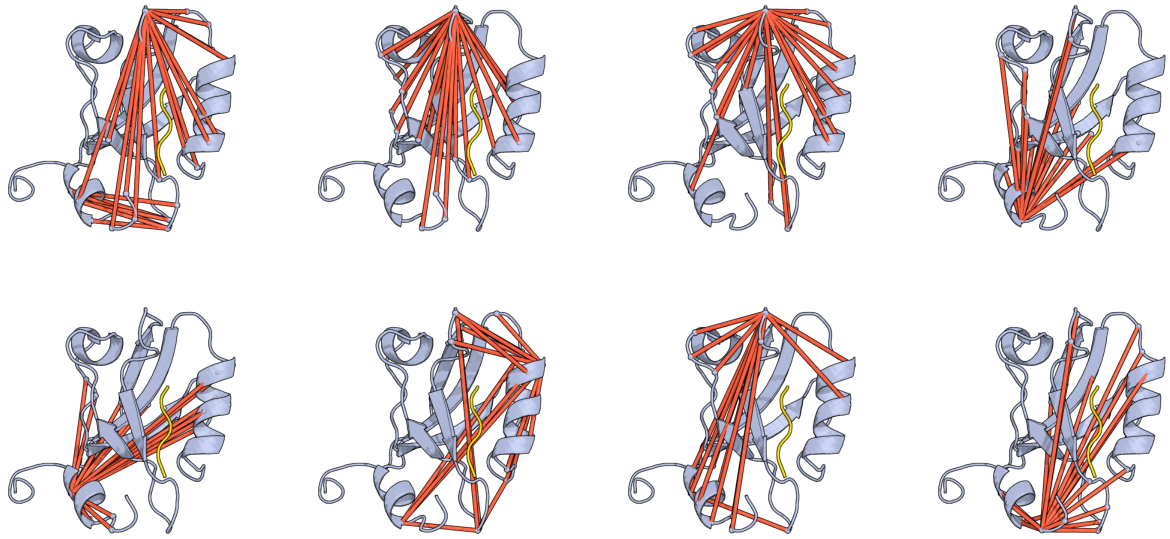

Figure S7: PDZ3WT – First 8 normal modes with the 20 C_α distances indicated that have the largest displacement per mode. Generally, each normal mode has one (or a few) anchor residues from which the largest displacements stretch out. Top row: NMA1-NMA4, bottom row: NMA5-NMA8.

4 PDZ3L6

MD simulations

For PDZ3L6, the only difference to the usual PDZ3 variant is the ligand that is longer by a single Lys residue (KKETWV). Considering the same *cis*-to-*trans* transition as before, we produced in total $89 \times 1\,\mu$s and $10 \times 10\,\mu$s long trajectories, which were not published before.

MoSAIC Clustering

With the contact criterion (10 % of all frames of a contact distance are below 0.45 nm) applied to the short trajectories, a total of 428 contact distances are derived. MoSAIC analysis was performed using the Pearson correlation, a resolution parameter of $\gamma = 0.4$ and every 5th frame of the short trajectories. A total of 12 clusters are derived, with the last 3 are again connected to the highly flexible N- and C-terminus. Thus, 9 clusters of dynamical interest are obtained where cluster C3, C5 and C6 are associated with closely connected clusters C3', C5', and C6', respectively. Due to their tertiary similarity and significant cross correlation, clusters and their primed associates are mostly discussed together in the main text.

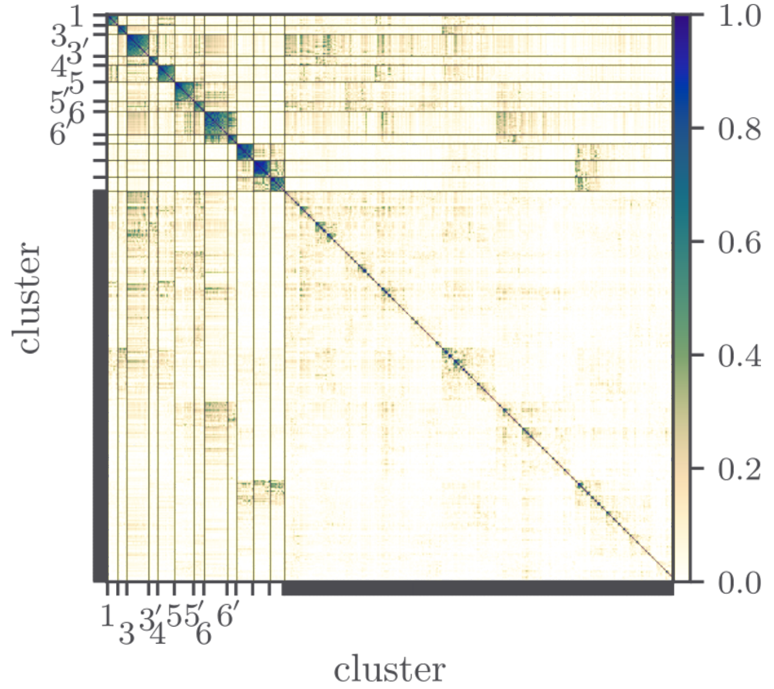

Figure S8: PDZ3L6 – MoSAIC analysis of 428 contact distances with a resolution parameter of $\gamma = 0.4$.

Table S6: PDZ3L6 – Contact cluster of dynamical interest.

**Cluster**	**Contacts**
C1	$r_{28,-5}$, $r_{73,-5}$, $r_{100,-5}$, $r_{101,-5}$, $r_{102,-5}$, $r_{103,-5}$, $r_{-5,-2}$
C2	$r_{28,100}$, $r_{29,100}$, $r_{30,100}$, $r_{35,100}$, $r_{37,100}$, $r_{97,100}$
C3	$r_{29,71}$, $r_{32,69}$, $r_{33,68}$, $r_{33,69}$, $r_{33,70}$, $r_{33,71}$, $r_{34,68}$, $r_{34,69}$, $r_{35,67}$, $r_{35,68}$, $r_{35,69}$, $r_{35,70}$, $r_{36,79}$, $r_{58,68}$, $r_{59,68}$, $r_{60,68}$
C3'	$r_{30,71}$, $r_{30,72}$, $r_{31,70}$, $r_{31,71}$, $r_{31,72}$, $r_{31,73}$
C4	$r_{27,-4}$, $r_{27,-3}$, $r_{28,-4}$, $r_{28,-3}$, $r_{29,-5}$, $r_{29,-4}$, $r_{30,-4}$, $r_{31,-5}$, $r_{31,-4}$, $r_{72,-4}$, $r_{72,-3}$, $r_{72,-2}$
C5	$r_{18,81}$, $r_{62,82}$, $r_{63,81}$, $r_{63,82}$, $r_{77,81}$, $r_{77,82}$, $r_{78,82}$, $r_{79,82}$, $r_{79,83}$, $r_{80,83}$, $r_{81,86}$, $r_{82,85}$, $r_{82,86}$, $r_{83,86}$
C5'	$r_{59,62}$, $r_{63,78}$, $r_{67,74}$, $r_{67,79}$, $r_{70,75}$, $r_{71,75}$, $r_{78,86}$
C6	$r_{18,82}$, $r_{18,83}$, $r_{18,86}$, $r_{18,-1}$, $r_{18,0}$, $r_{20,-1}$, $r_{21,24}$, $r_{21,25}$, $r_{21,42}$, $r_{21,43}$, $r_{21,45}$, $r_{21,46}$, $r_{21,-1}$, $r_{22,-1}$, $r_{24,46}$, $r_{24,0}$, $r_{25,0}$
C6'	$r_{17,22}$, $r_{18,21}$, $r_{19,22}$, $r_{19,46}$, $r_{19,84}$, $r_{21,0}$

C1

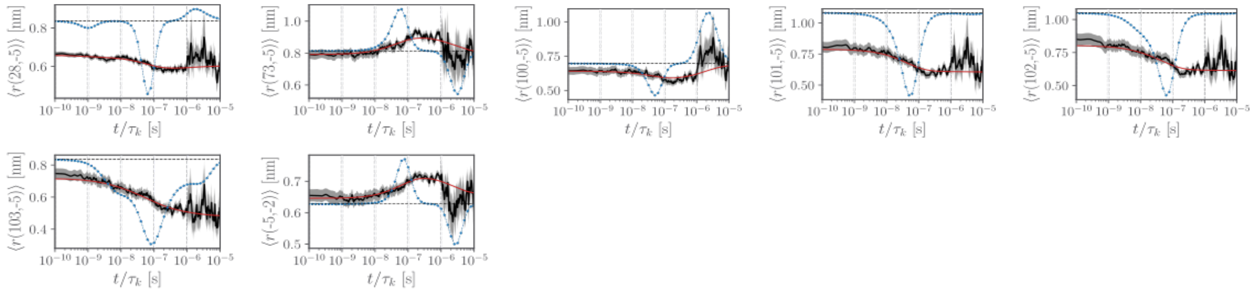
⟨r(28,-5)⟩ [nm]
⟨r(73,-5)⟩ [nm]
⟨r(100,-5)⟩ [nm]
⟨r(101,-5)⟩ [nm]
⟨r(102,-5)⟩ [nm]
⟨r(103,-5)⟩ [nm]
⟨r(-5,-2)⟩ [nm]
t/τ_k [s]

C2

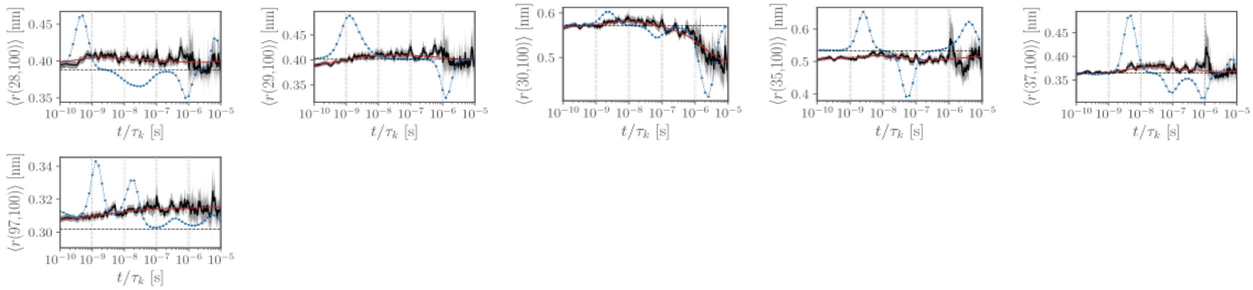
⟨r(28,100)⟩ [nm]
⟨r(29,100)⟩ [nm]
⟨r(30,100)⟩ [nm]
⟨r(35,100)⟩ [nm]
⟨r(37,100)⟩ [nm]
⟨r(97,100)⟩ [nm]
t/τ_k [s]

C3

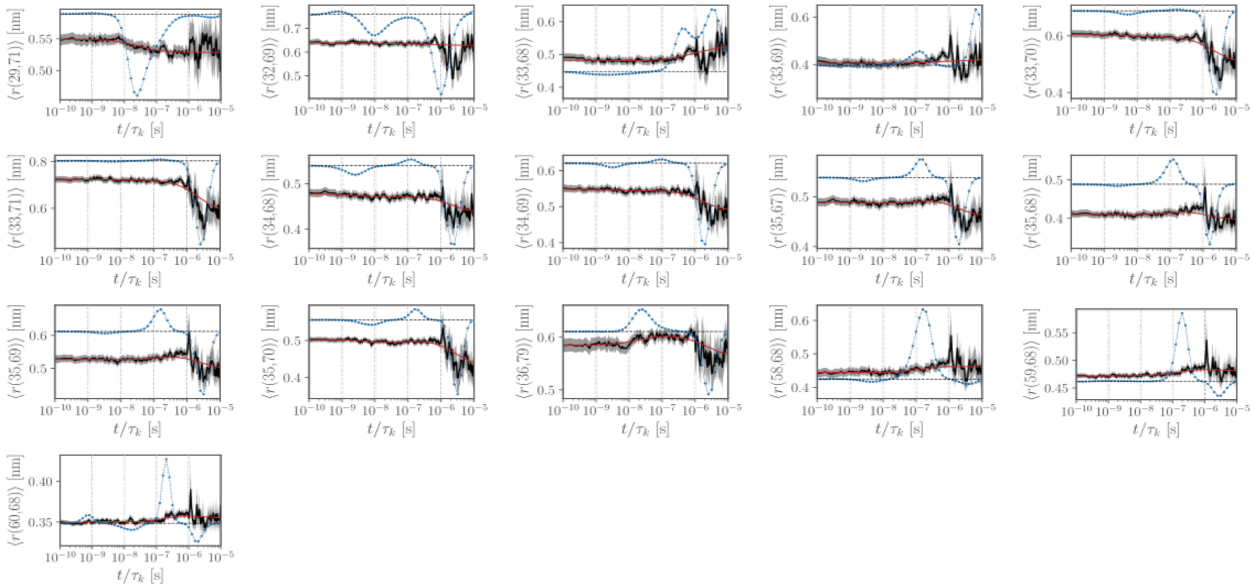
⟨r(29,71)⟩ [nm]
⟨r(32,69)⟩ [nm]
⟨r(33,68)⟩ [nm]
⟨r(33,69)⟩ [nm]
⟨r(33,70)⟩ [nm]
⟨r(33,71)⟩ [nm]
⟨r(34,68)⟩ [nm]
⟨r(34,69)⟩ [nm]
⟨r(35,67)⟩ [nm]
⟨r(35,68)⟩ [nm]
⟨r(35,69)⟩ [nm]
⟨r(35,70)⟩ [nm]
⟨r(36,79)⟩ [nm]
⟨r(58,68)⟩ [nm]
⟨r(59,68)⟩ [nm]
⟨r(60,68)⟩ [nm]
t/τ_k [s]

C3’

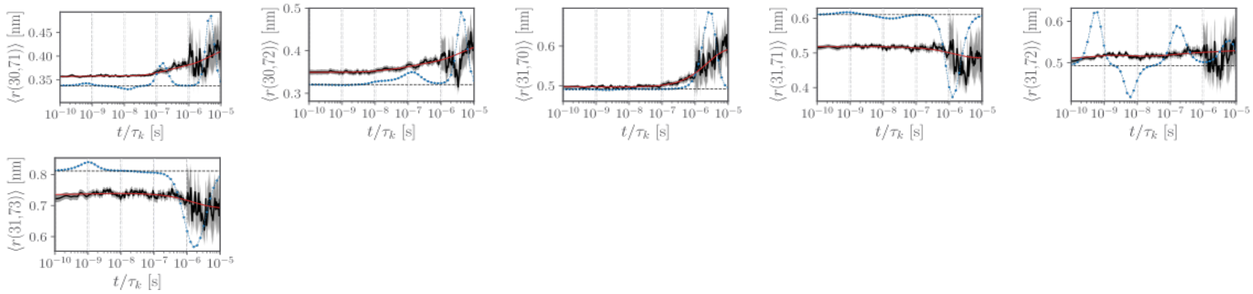
⟨r(30,71)⟩ [nm]
⟨r(30,72)⟩ [nm]
⟨r(31,70)⟩ [nm]
⟨r(31,71)⟩ [nm]
⟨r(31,72)⟩ [nm]
⟨r(31,73)⟩ [nm]
t/τ_k [s]

C4

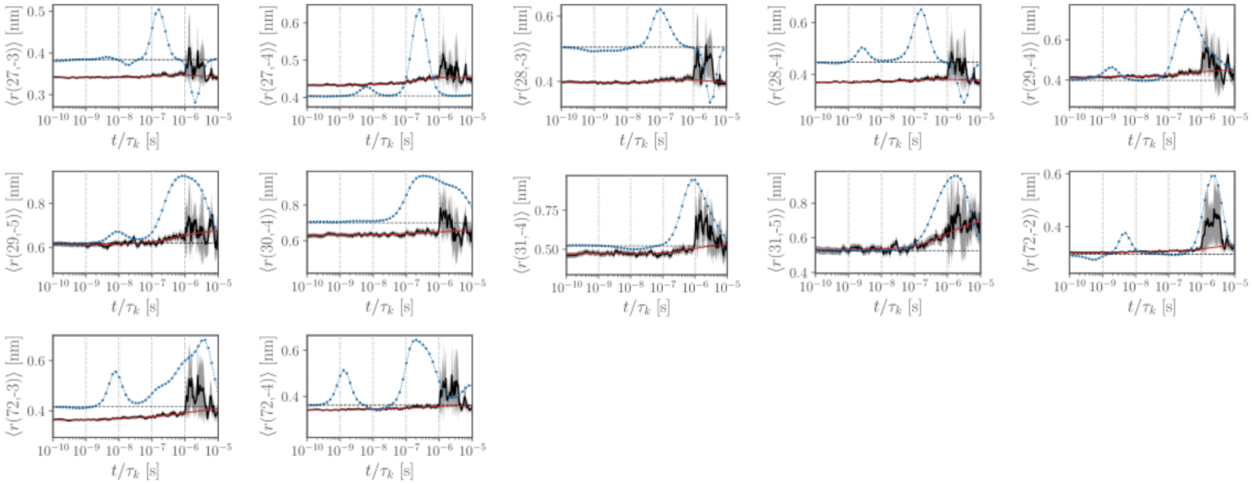

⟨r(27,-3)⟩ [nm]
⟨r(27,-4)⟩ [nm]
⟨r(28,-3)⟩ [nm]
⟨r(28,-4)⟩ [nm]
⟨r(29,-4)⟩ [nm]
⟨r(29,-5)⟩ [nm]
⟨r(30,-4)⟩ [nm]
⟨r(31,-4)⟩ [nm]
⟨r(31,-5)⟩ [nm]
⟨r(72,-2)⟩ [nm]
⟨r(72,-3)⟩ [nm]
⟨r(72,-4)⟩ [nm]
t/τ_k [s]

C5

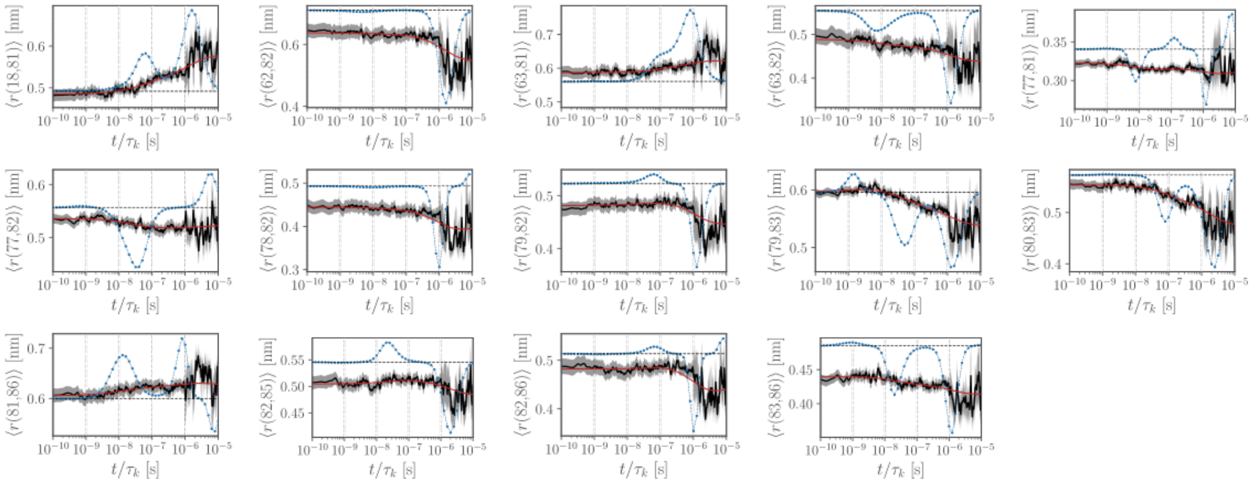

⟨r(18,81)⟩ [nm]
⟨r(62,82)⟩ [nm]
⟨r(63,81)⟩ [nm]
⟨r(63,82)⟩ [nm]
⟨r(77,81)⟩ [nm]
⟨r(77,82)⟩ [nm]
⟨r(78,82)⟩ [nm]
⟨r(79,82)⟩ [nm]
⟨r(79,83)⟩ [nm]
⟨r(80,83)⟩ [nm]
⟨r(81,86)⟩ [nm]
⟨r(82,85)⟩ [nm]
⟨r(82,86)⟩ [nm]
⟨r(83,86)⟩ [nm]
t/τ_k [s]

C5'

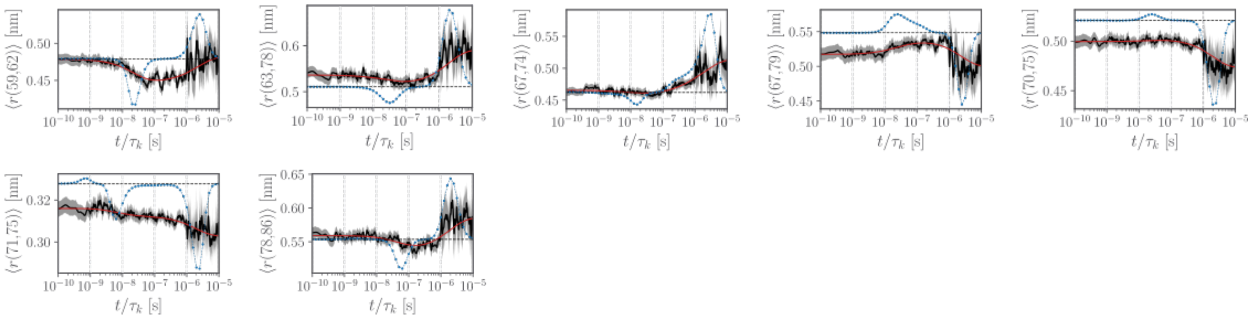

⟨r(59,62)⟩ [nm]
⟨r(63,78)⟩ [nm]
⟨r(67,74)⟩ [nm]
⟨r(67,79)⟩ [nm]
⟨r(70,75)⟩ [nm]
⟨r(71,75)⟩ [nm]
⟨r(78,86)⟩ [nm]
t/τ_k [s]

C6

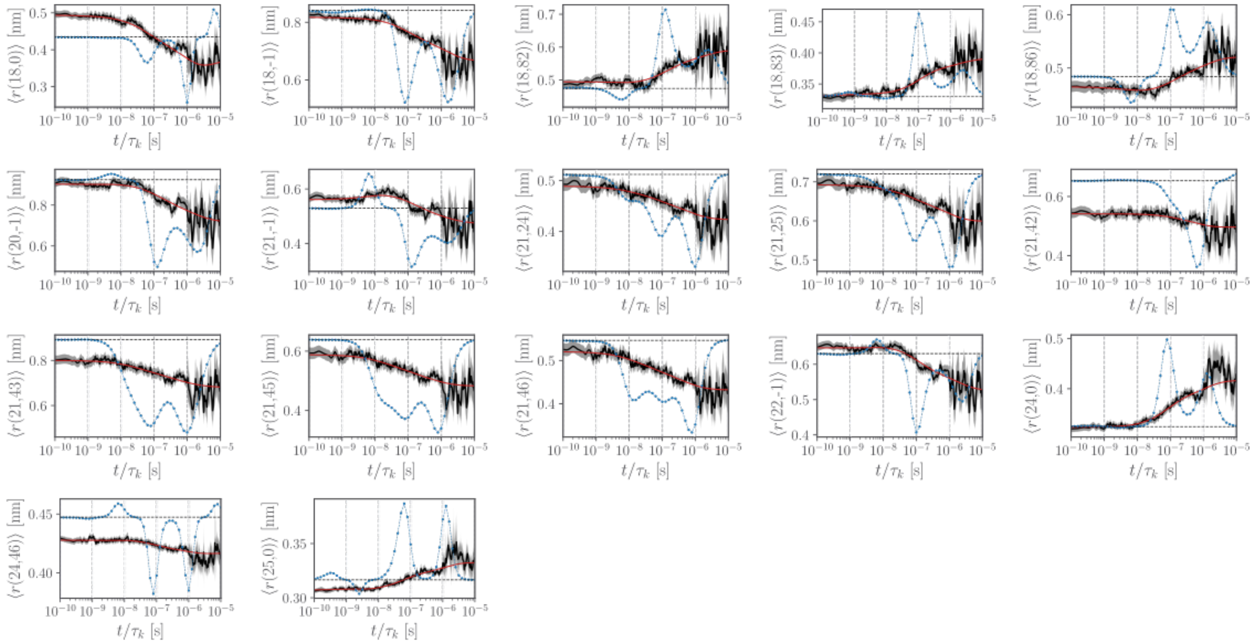

C6'

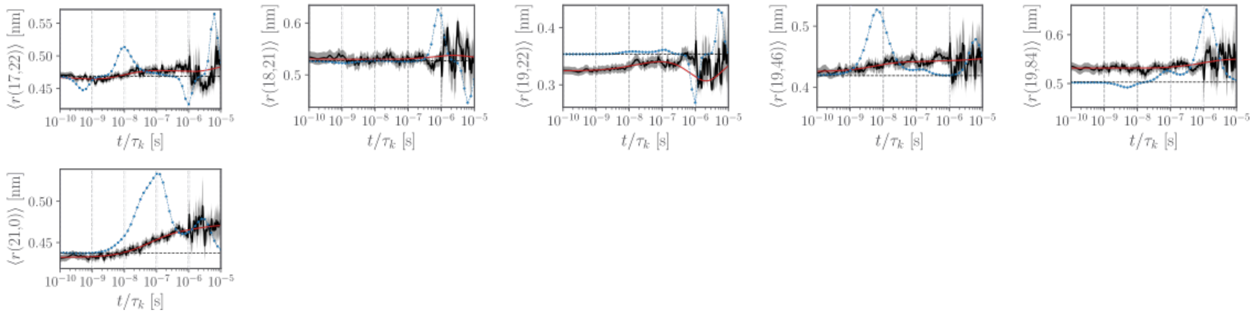

Figure S9: PDZ3L6 – Timescale analysis of each contact distance with $\lambda = 100$ and 10 fit parameters per order of magnitude.

5 PDZ2S

MD simulations

In PDZ2S, the photoswitch (anchored via residues Azo22 and Azo77) cross-links the binding pocket, i.e., there is no ligand in the binding pocket. Studying the *cis*-to-*trans* transition, Buchenberg et al.[S9] generated 100 nonequilibrium trajectories of 1 µs length, end extended 20 of them to 10 µs. Due to an unfortunate choice of these extended trajectories, many contact distances exhibit a significant jump at 1 µs. To avoid this problem, here we considered only the 20 long trajectories. As 6 out of these 20 runs lead to an unfolding of the α_2 helix (which is likely an artifact caused by the directly cross-linked photoswitch), these trajectories were discarded as well. Thus, we end up with 14×10 µs-long trajectories with a time step of $\Delta t = 20$ ps.

MoSAIC clustering

Using our contact criterion (10 % of the time the contact distance is below 0.45 nm) we obtain 330 contact distances for the MoSAIC analysis. As a similarity measure, the normalized mutual information as introduced by Nagel et al.[S10] provided the most reproducible results in this highly perturbed system — which also requires a low resolution parameter of $\gamma = 0.1$. This yielded 7 clusters with two of them being dominated by N- and C-terminus residues, which are neglected in the analysis. Note, due to the much more disruptive intrusion of the photoswitch, the respective clusters show a relatively large cross-correlation. Nonetheless, the 5 remaining clusters can be well separated into the tertiary regions as before.

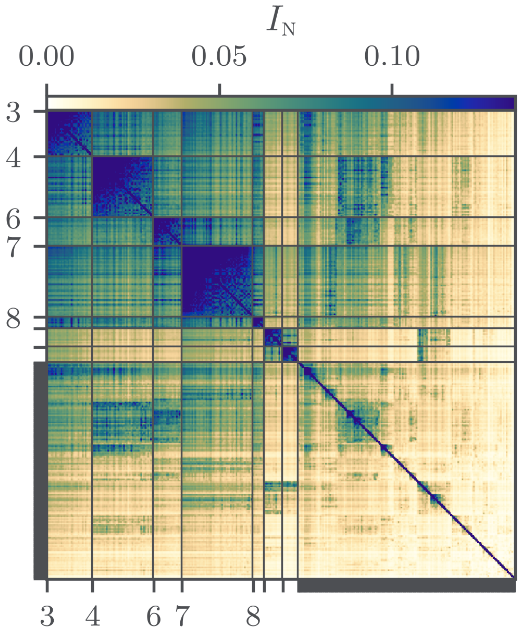

Figure S10: PDZ2S – MoSAIC analysis of 330 contact distances, using a resolution parameter of $\gamma = 0.1$. Here, we employed the normalized mutual information as similarity measure instead of the usual Pearson correlation.

Table S7: PDZ2S – Contact cluster of dynamical interest.

**Cluster**	**Contacts**
C3	$r_{24,35}$, $r_{24,36}$, $r_{24,37}$, $r_{25,28}$, $r_{25,35}$, $r_{25,36}$, $r_{25,37}$, $r_{26,29}$, $r_{26,31}$, $r_{26,34}$, $r_{26,35}$, $r_{26,36}$, $r_{26,37}$, $r_{26,72}$, $r_{27,30}$, $r_{27,31}$, $r_{27,33}$, $r_{27,34}$, $r_{27,72}$, $r_{28,31}$, $r_{28,32}$, $r_{28,33}$, $r_{28,34}$, $r_{28,37}$, $r_{28,67}$, $r_{29,32}$, $r_{31,34}$, $r_{31,35}$, $r_{31,37}$, $r_{31,58}$, $r_{33,37}$, $r_{34,37}$
C4	$r_{23,72}$, $r_{24,72}$, $r_{25,72}$, $r_{35,70}$, $r_{36,70}$, $r_{36,71}$, $r_{36,75}$, $r_{36,76}$, $r_{62,75}$, $r_{62,82}$, $r_{63,78}$, $r_{65,71}$, $r_{65,74}$, $r_{65,75}$, $r_{66,70}$, $r_{66,71}$, $r_{66,72}$, $r_{66,75}$, $r_{67,70}$, $r_{67,72}$, $r_{67,75}$, $r_{67,76}$, $r_{67,78}$, $r_{67,79}$, $r_{68,71}$, $r_{68,72}$, $r_{69,72}$, $r_{70,73}$, $r_{70,74}$, $r_{70,75}$, $r_{71,74}$, $r_{71,75}$, $r_{72,75}$, $r_{72,76}$, $r_{73,77}$, $r_{74,77}$, $r_{74,78}$, $r_{75,78}$, $r_{75,79}$, $r_{76,80}$, $r_{78,82}$, $r_{82,85}$, $r_{82,86}$
C6	$r_{12,19}$, $r_{13,18}$, $r_{13,19}$, $r_{14,17}$, $r_{14,18}$, $r_{14,19}$, $r_{14,20}$, $r_{15,18}$, $r_{15,19}$, $r_{15,83}$, $r_{16,19}$, $r_{16,83}$, $r_{17,22}$, $r_{17,77}$, $r_{18,22}$, $r_{19,22}$, $r_{19,77}$, $r_{19,86}$, $r_{19,88}$, $r_{21,38}$
C7	$r_{9,52}$, $r_{10,50}$, $r_{10,52}$, $r_{11,50}$, $r_{11,52}$, $r_{12,46}$, $r_{12,47}$, $r_{12,49}$, $r_{12,50}$, $r_{13,46}$, $r_{19,45}$, $r_{19,46}$, $r_{19,47}$, $r_{20,40}$, $r_{20,41}$, $r_{20,42}$, $r_{20,45}$, $r_{20,46}$, $r_{20,47}$, $r_{21,39}$, $r_{21,40}$, $r_{21,46}$, $r_{21,47}$, $r_{22,40}$, $r_{22,42}$, $r_{38,41}$, $r_{40,53}$, $r_{40,54}$, $r_{41,47}$, $r_{41,48}$, $r_{41,53}$, $r_{41,54}$, $r_{42,45}$, $r_{42,48}$, $r_{43,48}$, $r_{44,48}$, $r_{44,49}$, $r_{45,48}$, $r_{45,49}$, $r_{46,49}$, $r_{46,50}$, $r_{46,53}$, $r_{47,50}$, $r_{47,51}$, $r_{47,52}$, $r_{47,53}$, $r_{48,51}$, $r_{49,52}$, $r_{50,53}$, $r_{51,54}$
C8	$r_{22,37}$, $r_{22,38}$, $r_{22,39}$, $r_{23,37}$, $r_{23,38}$, $r_{23,39}$, $r_{24,39}$, $r_{24,77}$

C3

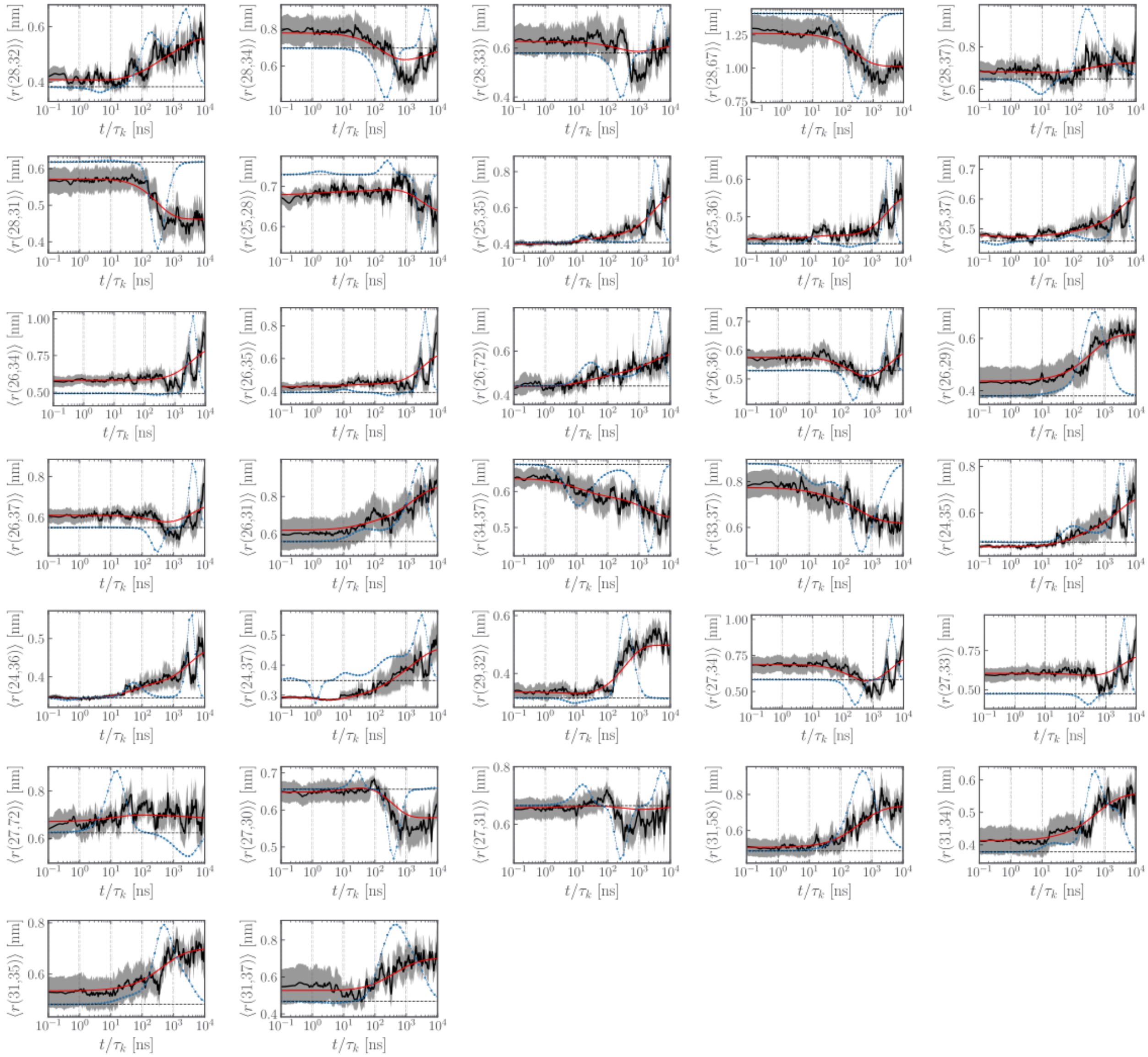

⟨r(28,32)⟩ [nm]
⟨r(28,34)⟩ [nm]
⟨r(28,33)⟩ [nm]
⟨r(28,67)⟩ [nm]
⟨r(28,37)⟩ [nm]
⟨r(28,31)⟩ [nm]
⟨r(25,28)⟩ [nm]
⟨r(25,35)⟩ [nm]
⟨r(25,36)⟩ [nm]
⟨r(25,37)⟩ [nm]
⟨r(26,34)⟩ [nm]
⟨r(26,35)⟩ [nm]
⟨r(26,72)⟩ [nm]
⟨r(26,36)⟩ [nm]
⟨r(26,29)⟩ [nm]
⟨r(26,37)⟩ [nm]
⟨r(26,31)⟩ [nm]
⟨r(34,37)⟩ [nm]
⟨r(33,37)⟩ [nm]
⟨r(24,35)⟩ [nm]
⟨r(24,36)⟩ [nm]
⟨r(24,37)⟩ [nm]
⟨r(29,32)⟩ [nm]
⟨r(27,34)⟩ [nm]
⟨r(27,33)⟩ [nm]
⟨r(27,72)⟩ [nm]
⟨r(27,30)⟩ [nm]
⟨r(27,31)⟩ [nm]
⟨r(31,58)⟩ [nm]
⟨r(31,34)⟩ [nm]
⟨r(31,35)⟩ [nm]
⟨r(31,37)⟩ [nm]
t/τk [ns]

C4

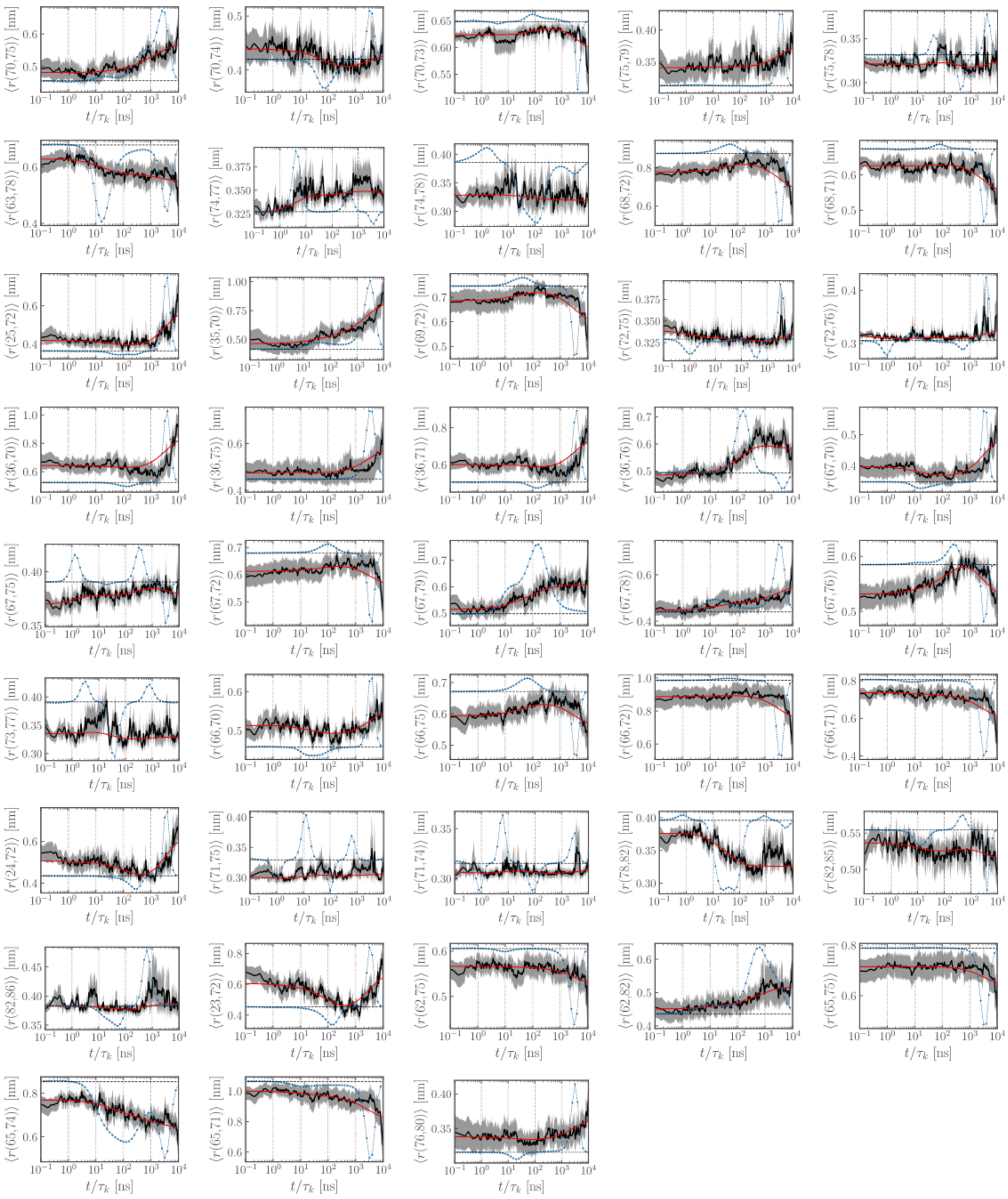
⟨r(70,75)⟩ [nm]
⟨r(70,74)⟩ [nm]
⟨r(70,73)⟩ [nm]
⟨r(75,79)⟩ [nm]
⟨r(75,78)⟩ [nm]
⟨r(63,78)⟩ [nm]
⟨r(74,77)⟩ [nm]
⟨r(74,78)⟩ [nm]
⟨r(68,72)⟩ [nm]
⟨r(68,71)⟩ [nm]
⟨r(25,72)⟩ [nm]
⟨r(35,70)⟩ [nm]
⟨r(69,72)⟩ [nm]
⟨r(72,75)⟩ [nm]
⟨r(72,76)⟩ [nm]
⟨r(36,70)⟩ [nm]
⟨r(36,75)⟩ [nm]
⟨r(36,71)⟩ [nm]
⟨r(36,76)⟩ [nm]
⟨r(67,70)⟩ [nm]
⟨r(67,75)⟩ [nm]
⟨r(67,72)⟩ [nm]
⟨r(67,79)⟩ [nm]
⟨r(67,78)⟩ [nm]
⟨r(67,76)⟩ [nm]
⟨r(73,77)⟩ [nm]
⟨r(66,70)⟩ [nm]
⟨r(66,75)⟩ [nm]
⟨r(66,72)⟩ [nm]
⟨r(66,71)⟩ [nm]
⟨r(24,72)⟩ [nm]
⟨r(71,75)⟩ [nm]
⟨r(71,74)⟩ [nm]
⟨r(78,82)⟩ [nm]
⟨r(82,85)⟩ [nm]
⟨r(82,86)⟩ [nm]
⟨r(23,72)⟩ [nm]
⟨r(62,75)⟩ [nm]
⟨r(62,82)⟩ [nm]
⟨r(65,75)⟩ [nm]
⟨r(65,74)⟩ [nm]
⟨r(65,71)⟩ [nm]
⟨r(76,80)⟩ [nm]
t/τk [ns]

C6

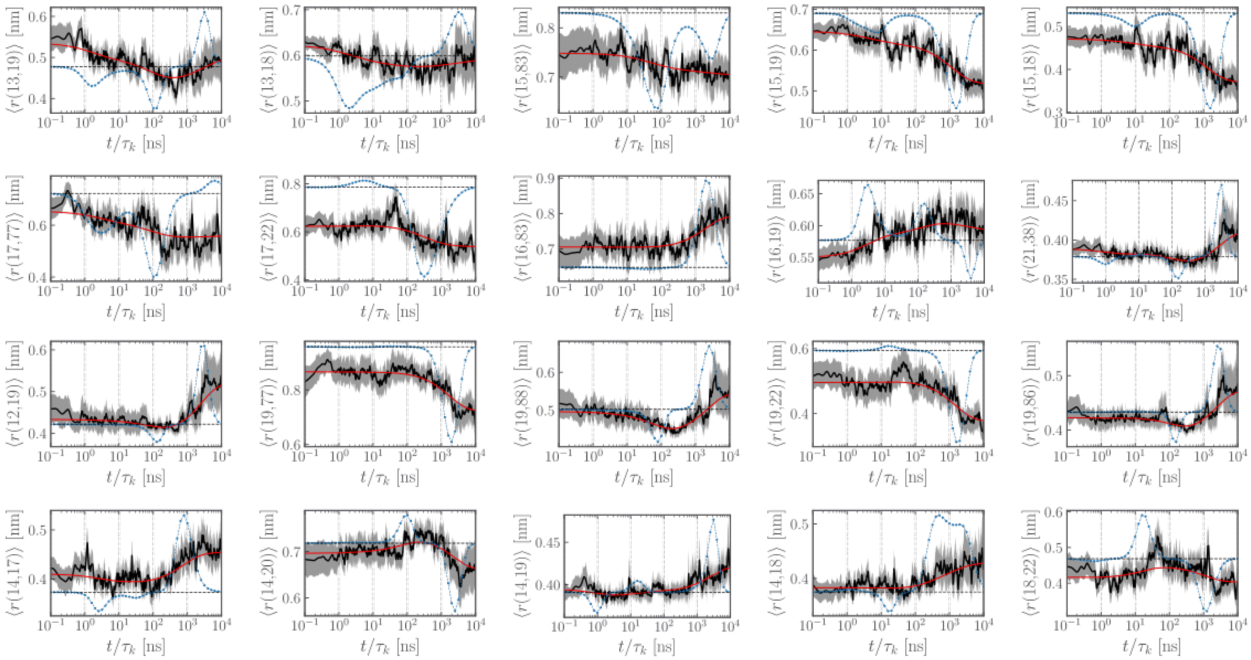
⟨r(13,19)⟩ [nm]
⟨r(13,18)⟩ [nm]
⟨r(15,83)⟩ [nm]
⟨r(15,19)⟩ [nm]
⟨r(15,18)⟩ [nm]
⟨r(17,77)⟩ [nm]
⟨r(17,22)⟩ [nm]
⟨r(16,83)⟩ [nm]
⟨r(16,19)⟩ [nm]
⟨r(21,38)⟩ [nm]
⟨r(12,19)⟩ [nm]
⟨r(19,77)⟩ [nm]
⟨r(19,88)⟩ [nm]
⟨r(19,22)⟩ [nm]
⟨r(19,86)⟩ [nm]
⟨r(14,17)⟩ [nm]
⟨r(14,20)⟩ [nm]
⟨r(14,19)⟩ [nm]
⟨r(14,18)⟩ [nm]
⟨r(18,22)⟩ [nm]
t/τk [ns]

C8

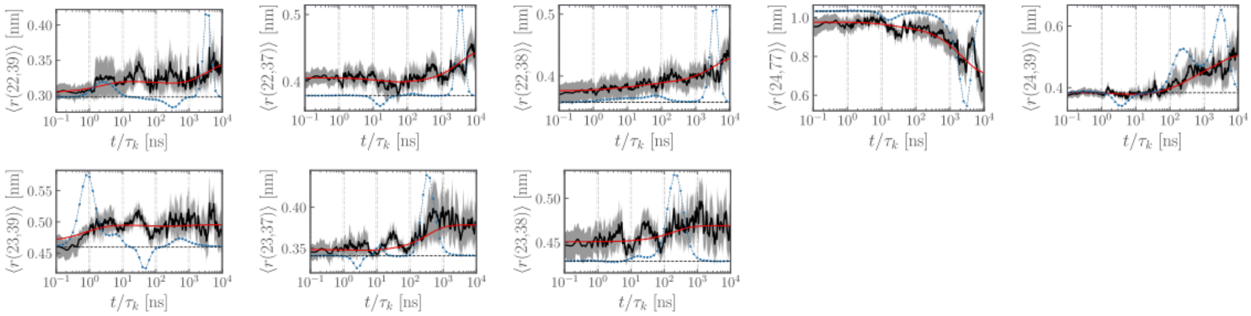
⟨r(22,39)⟩ [nm]
⟨r(22,37)⟩ [nm]
⟨r(22,38)⟩ [nm]
⟨r(24,77)⟩ [nm]
⟨r(24,39)⟩ [nm]
⟨r(23,39)⟩ [nm]
⟨r(23,37)⟩ [nm]
⟨r(23,38)⟩ [nm]
t/τk [ns]

C7

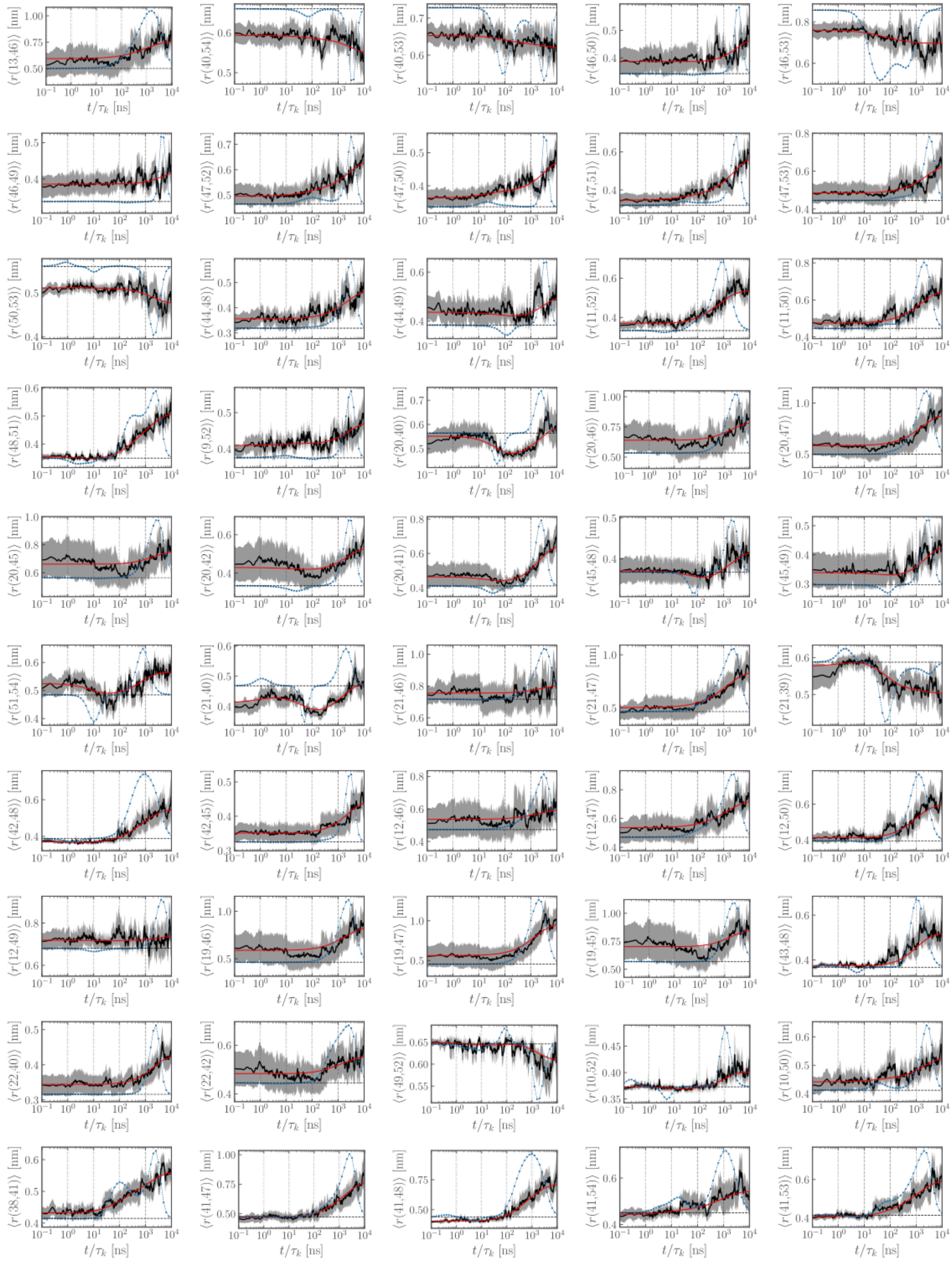

Figure S11: PDZ2S – Timescale analysis of each contact distance with $\lambda = 50$ and 10 fit parameters per order of magnitude.

6 PDZ2L

MD simulations

In PDZ2L the photoswitch is attached to the ligand (at Azo(-1) and Azo(-6)). Performing a *trans*-to-*cis* transitions results in a squeezing of ligand and its subsequent detachment from the binding pocket. Bozovic et al.[S11] produced $100 \times 1\,\mu$s long trajectories with an time step of $\Delta t = 20$ ps, 19 of it were extended to $10\,\mu$s. This yields a data set of $80 \times 1\,\mu$s and $19 \times 10\,\mu$s-long simulations.

MoSAIC analysis Using our contact distance criterion (10 % of all simulated frames must be below 0.45 nm) on the short trajectories, we obtained a total of 441 contact distances, which are used in the MoSAIC analysis. A resolution parameter of $\gamma = 0.6$ is chosen, with all frames utilized and the Pearson correlation as similarity measure.

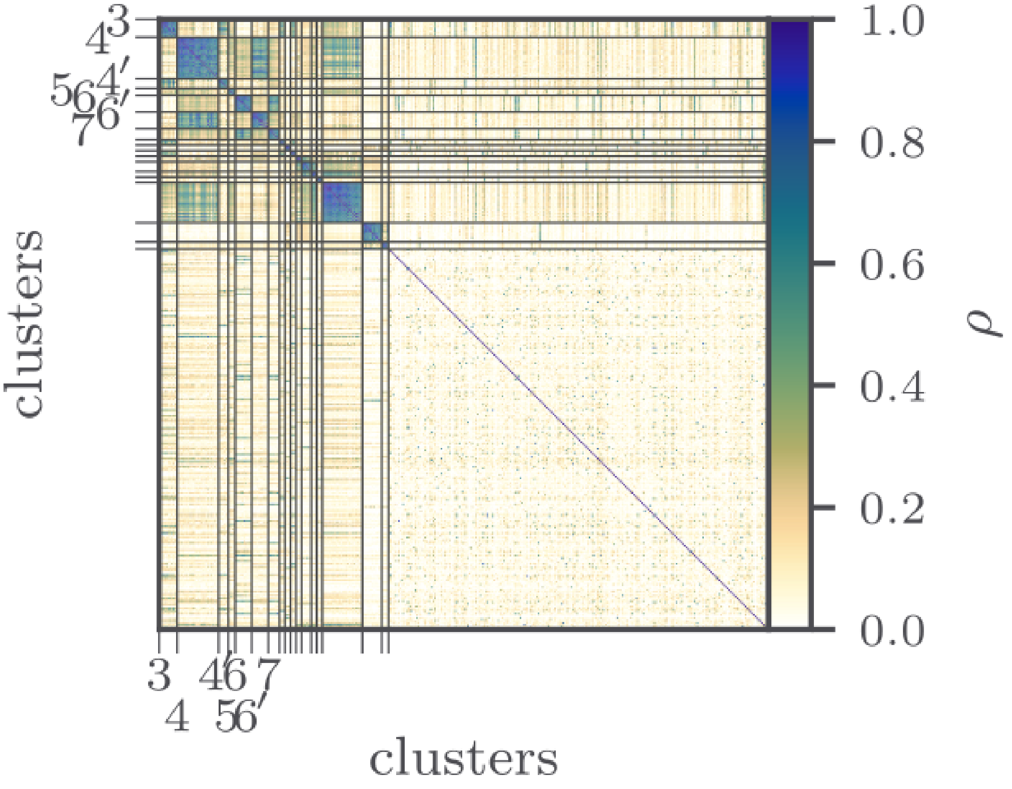

Figure S12: PDZ2L – MoSAIC analysis of 441 contact distances with a resolution parameter of $\gamma = 0.6$.

While we obtained in total 17 contact cluster, several of them are dominated by highly flexible regions such as the N-terminus or the tail of the ligand (which is not bound in the binding pocket). As these motion are of little functional interest, we remove them and end up with 7 relevant contact clusters, where the clusters C4 and C4' as well as C6 and C6' are at similar tertiary regions.

Table S8: PDZ2L – Contact cluster of dynamical interest.

**Cluster**	**Contacts**
C3	$r_{22,74}$, $r_{24,70}$, $r_{25,35}$, $r_{25,69}$, $r_{25,70}$, $r_{26,67}$, $r_{26,68}$, $r_{26,69}$, $r_{26,70}$, $r_{27,70}$, $r_{35,69}$, $r_{35,70}$, $r_{35,75}$
C4	$r_{20,-2}$, $r_{21,-3}$, $r_{21,-2}$, $r_{21,-1}$, $r_{21,0}$, $r_{22,-4}$, $r_{22,-3}$, $r_{22,-2}$, $r_{22,-1}$, $r_{23,-4}$, $r_{23,-3}$, $r_{24,-4}$, $r_{38,-4}$, $r_{38,-3}$, $r_{71,-7}$, $r_{71,-6}$, $r_{71,-5}$, $r_{71,-4}$, $r_{71,-3}$, $r_{71,-2}$, $r_{71,-1}$, $r_{72,-7}$, $r_{72,-6}$, $r_{72,-5}$, $r_{72,-2}$, $r_{72,-1}$, $r_{75,-2}$, $r_{75,-1}$, $r_{76,-1}$, $r_{79,-1}$
C4'	$r_{22,71}$, $r_{22,75}$, $r_{23,71}$, $r_{24,71}$, $r_{35,71}$, $r_{35,74}$, $r_{66,69}$
C5	$r_{13,78}$, $r_{22,78}$, $r_{35,78}$, $r_{66,78}$, $r_{78,85}$
C6	$r_{11,49}$, $r_{12,45}$, $r_{13,45}$, $r_{14,45}$, $r_{17,45}$, $r_{18,44}$, $r_{18,45}$, $r_{18,46}$, $r_{19,44}$, $r_{19,45}$, $r_{19,46}$, $r_{20,46}$
C6'	$r_{16,-1}$, $r_{17,-1}$, $r_{17,0}$, $r_{18,-1}$, $r_{19,0}$, $r_{20,-1}$, $r_{20,0}$, $r_{41,0}$, $r_{75,-6}$, $r_{76,-6}$, $r_{78,-1}$, $r_{79,-6}$
C7	$r_{40,46}$, $r_{40,47}$, $r_{41,47}$, $r_{42,47}$, $r_{44,47}$, $r_{44,48}$, $r_{46,51}$, $r_{46,52}$

C3

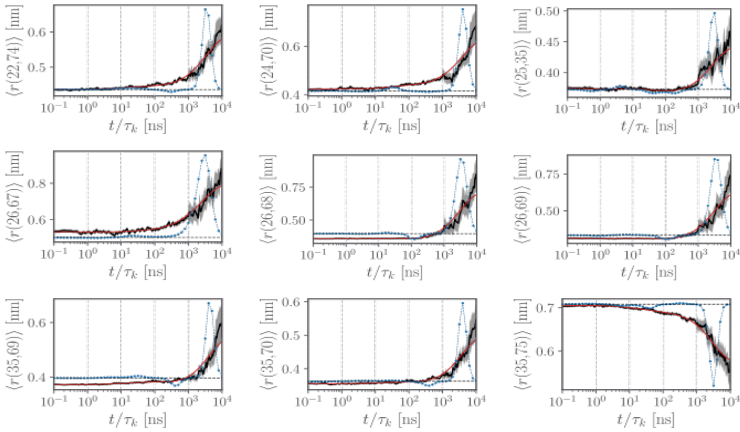

C4

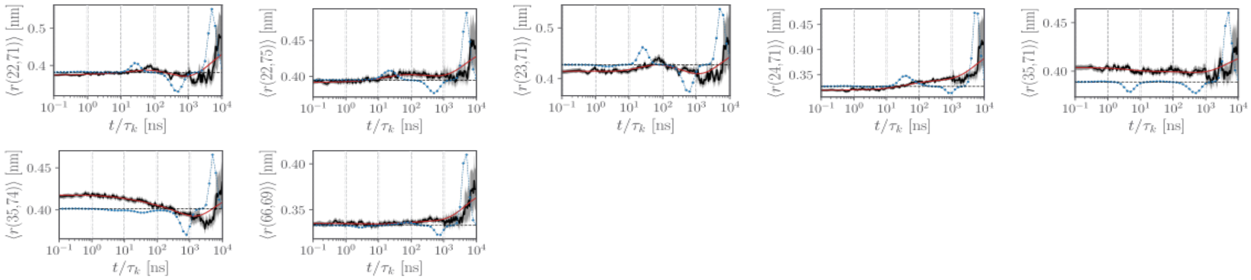

C4’

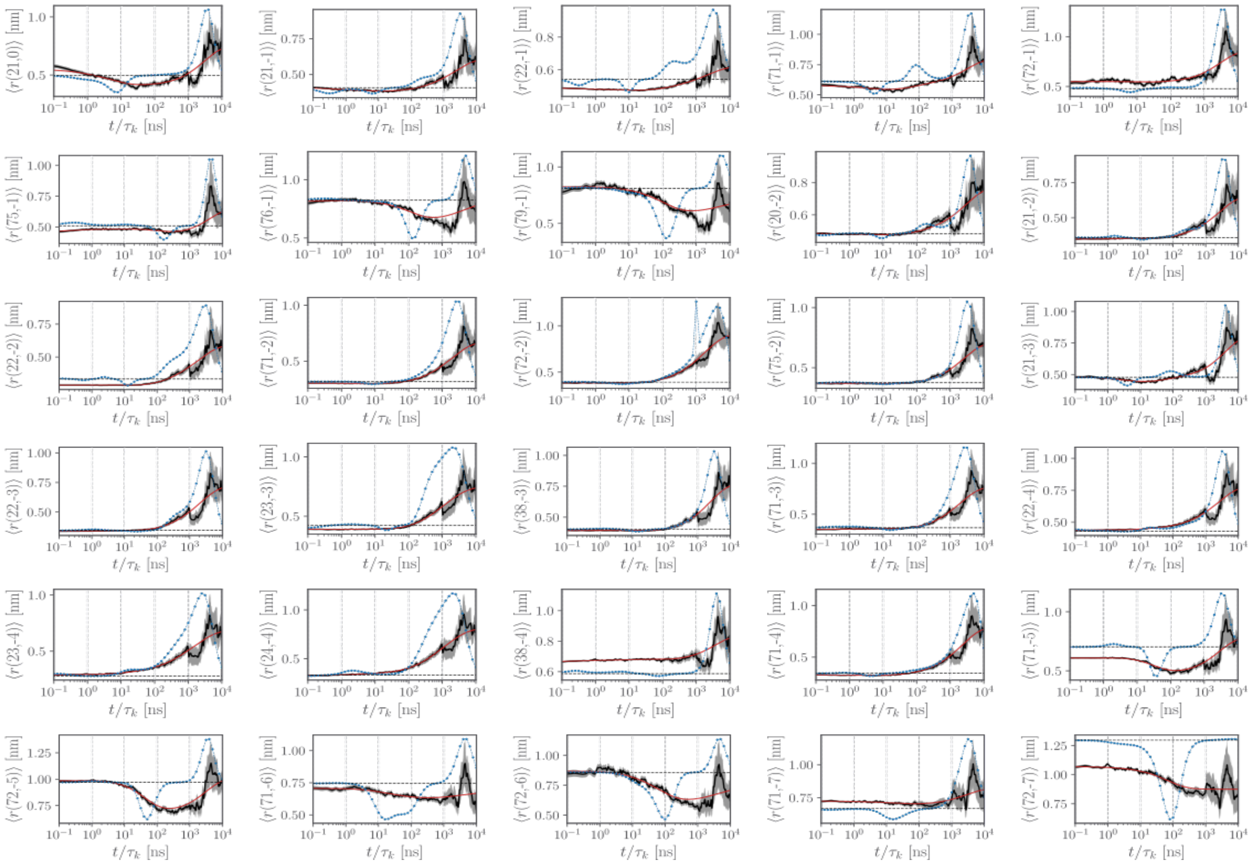

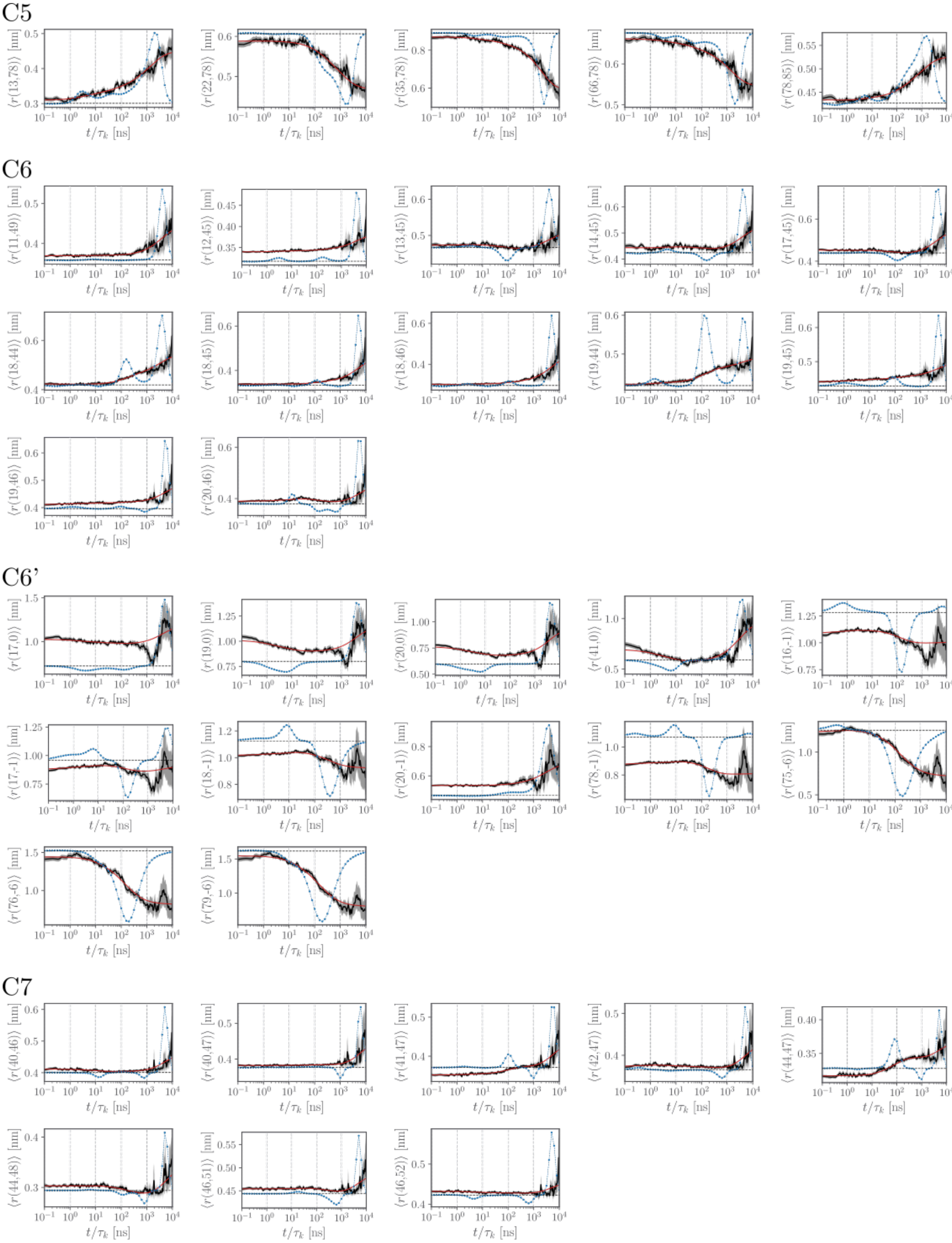

Figure S13: PDZ2L – Timescale analysis of each contact distance with $\lambda = 200$ and 10 fit parameters per order of magnitude.

Cis-to-*trans* transition

Bozovic et al.[S11] also studied the *cis*-to-*trans* transition of PDZ2L, corresponding to the binding of the ligand. They produced $10 \times 10\,\mu$s and $100 \times 1\,\mu$s long trajectories with a time step of $\Delta t = 20$ ps. As usual, we employed MoSAIC clustering to all contact distances (a total of 403) that fulfill the 10 % criterion for the 1 μs data set, utilizing a resolution parameter of $\gamma = 0.4$.

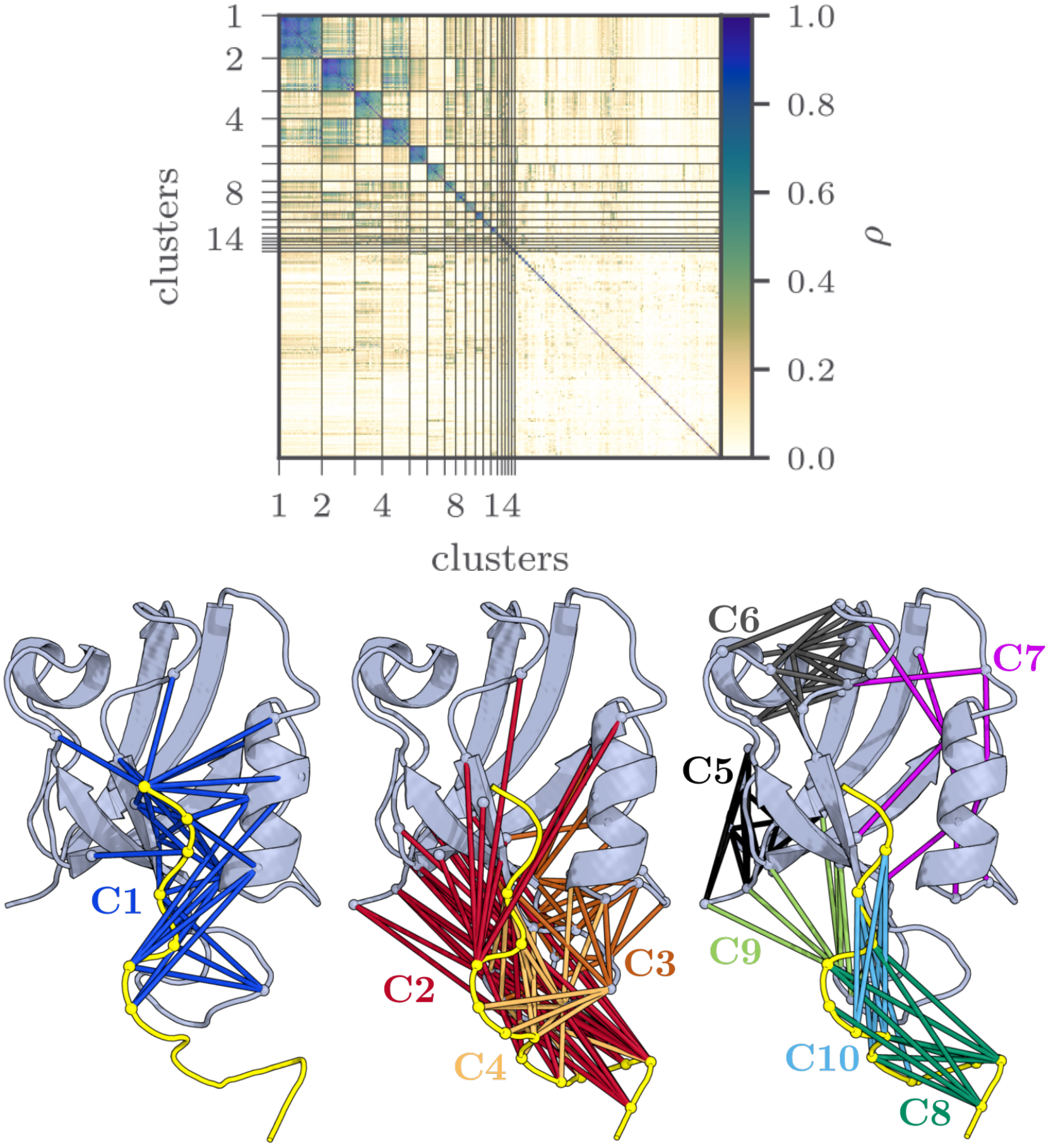

Figure S14: PDZ2L *cis*-to-*trans* – MoSAIC analysis with $\gamma = 0.4$ and the resulting clusters indicated.

For each averaged distance a timescale analysis is employed with $\lambda = 100$ and we derive the dynamical contents for each cluster as well as all of them combined, see Fig. S15.

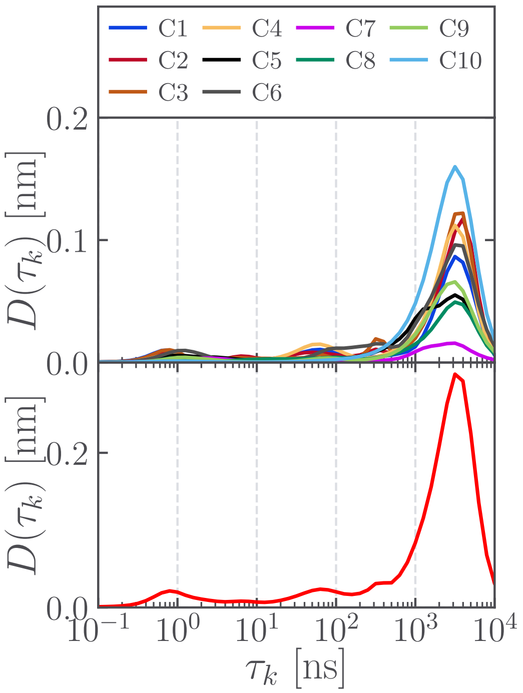

Figure S15: PDZ2L *cis*-to-*trans* – Dynamical content of each cluster and all of them combined, using $\lambda = 100$ and 10 fit parameters per order of magnitude.

Table S9: PDZ2L *cis*-to-*trans* – Contact cluster of dynamical interest.

**Cluster**	**Contacts**
C1	$r_{17,-1}$, $r_{20,-2}$, $r_{20,-1}$, $r_{21,-3}$, $r_{21,-2}$, $r_{21,-1}$, $r_{21,0}$, $r_{22,-4}$, $r_{22,-3}$, $r_{22,-2}$, $r_{22,-1}$, $r_{23,-4}$, $r_{23,-3}$, $r_{24,-4}$, $r_{27,-6}$, $r_{27,-5}$, $r_{27,-4}$, $r_{38,-3}$, $r_{38,0}$, $r_{41,-1}$, $r_{41,0}$, $r_{71,-7}$, $r_{71,-6}$, $r_{71,-5}$, $r_{71,-4}$, $r_{71,-3}$, $r_{71,-2}$, $r_{71,-1}$, $r_{72,-7}$, $r_{72,-6}$, $r_{72,-2}$, $r_{72,-1}$, $r_{75,-6}$, $r_{75,-2}$, $r_{75,-1}$, $r_{76,-6}$, $r_{76,-1}$, $r_{78,-1}$, $r_{79,-1}$
C2	$r_{5,-15}$, $r_{17,-6}$, $r_{20,-6}$, $r_{21,-6}$, $r_{22,-6}$, $r_{23,-10}$, $r_{31,-14}$, $r_{31,-13}$, $r_{31,-11}$, $r_{31,-10}$, $r_{36,-14}$, $r_{36,-11}$, $r_{38,-10}$, $r_{54,-14}$, $r_{55,-14}$, $r_{55,-13}$, $r_{56,-15}$, $r_{78,-6}$, $r_{79,-6}$, $r_{90,-14}$, $r_{91,-15}$, $r_{92,-15}$, $r_{92,-14}$, $r_{92,-13}$, $r_{93,-15}$, $r_{93,-14}$, $r_{93,-13}$, $r_{93,-12}$, $r_{94,-15}$, $r_{94,-14}$
C3	$r_{22,74}$, $r_{23,27}$, $r_{23,34}$, $r_{24,34}$, $r_{24,70}$, $r_{25,33}$, $r_{25,34}$, $r_{25,35}$, $r_{25,69}$, $r_{25,70}$, $r_{26,30}$, $r_{26,33}$, $r_{26,68}$, $r_{26,69}$, $r_{26,70}$, $r_{27,70}$, $r_{27,71}$, $r_{28,36}$, $r_{33,57}$, $r_{34,66}$, $r_{34,67}$, $r_{35,69}$, $r_{35,70}$, $r_{35,75}$, $r_{35,78}$
C4	$r_{23,-9}$, $r_{24,-9}$, $r_{27,-9}$, $r_{27,-8}$, $r_{27,-7}$, $r_{28,-11}$, $r_{28,-9}$, $r_{28,-7}$, $r_{28,-5}$, $r_{28,-4}$, $r_{28,70}$, $r_{28,71}$, $r_{29,-15}$, $r_{29,-14}$, $r_{29,-12}$, $r_{29,-11}$, $r_{29,-10}$, $r_{29,-9}$, $r_{29,-8}$, $r_{29,-5}$, $r_{29,-4}$, $r_{30,-9}$, $r_{30,-4}$, $r_{31,-4}$, $r_{32,-14}$
C5	$r_{1,91}$, $r_{1,93}$, $r_{1,94}$, $r_{2,91}$, $r_{2,92}$, $r_{2,93}$, $r_{2,94}$, $r_{3,90}$, $r_{3,91}$, $r_{3,92}$, $r_{3,93}$, $r_{3,94}$, $r_{4,90}$, $r_{4,91}$, $r_{4,92}$, $r_{4,93}$
C6	$r_{9,51}$, $r_{10,51}$, $r_{11,49}$, $r_{11,51}$, $r_{12,45}$, $r_{13,45}$, $r_{14,43}$, $r_{14,44}$, $r_{14,45}$, $r_{17,45}$, $r_{18,44}$, $r_{18,45}$, $r_{18,46}$, $r_{19,44}$, $r_{19,45}$, $r_{20,46}$
C7	$r_{13,78}$, $r_{18,81}$, $r_{22,78}$, $r_{35,74}$, $r_{66,77}$, $r_{66,78}$, $r_{69,74}$, $r_{70,74}$, $r_{77,81}$, $r_{78,85}$
C8	$r_{-15,-10}$, $r_{-15,-7}$, $r_{-15,-6}$, $r_{-15,-5}$, $r_{-14,-10}$, $r_{-14,-9}$, $r_{-14,-6}$, $r_{-14,-5}$, $r_{-13,-9}$
C9	$r_{24,30}$, $r_{30,34}$, $r_{30,36}$, $r_{31,36}$, $r_{31,55}$, $r_{31,57}$, $r_{31,90}$, $r_{31,93}$, $r_{31,94}$
C10	$r_{-11,-4}$, $r_{-10,-4}$, $r_{-10,-3}$, $r_{-9,-4}$, $r_{-9,-3}$, $r_{-8,-4}$, $r_{-8,-3}$

C1

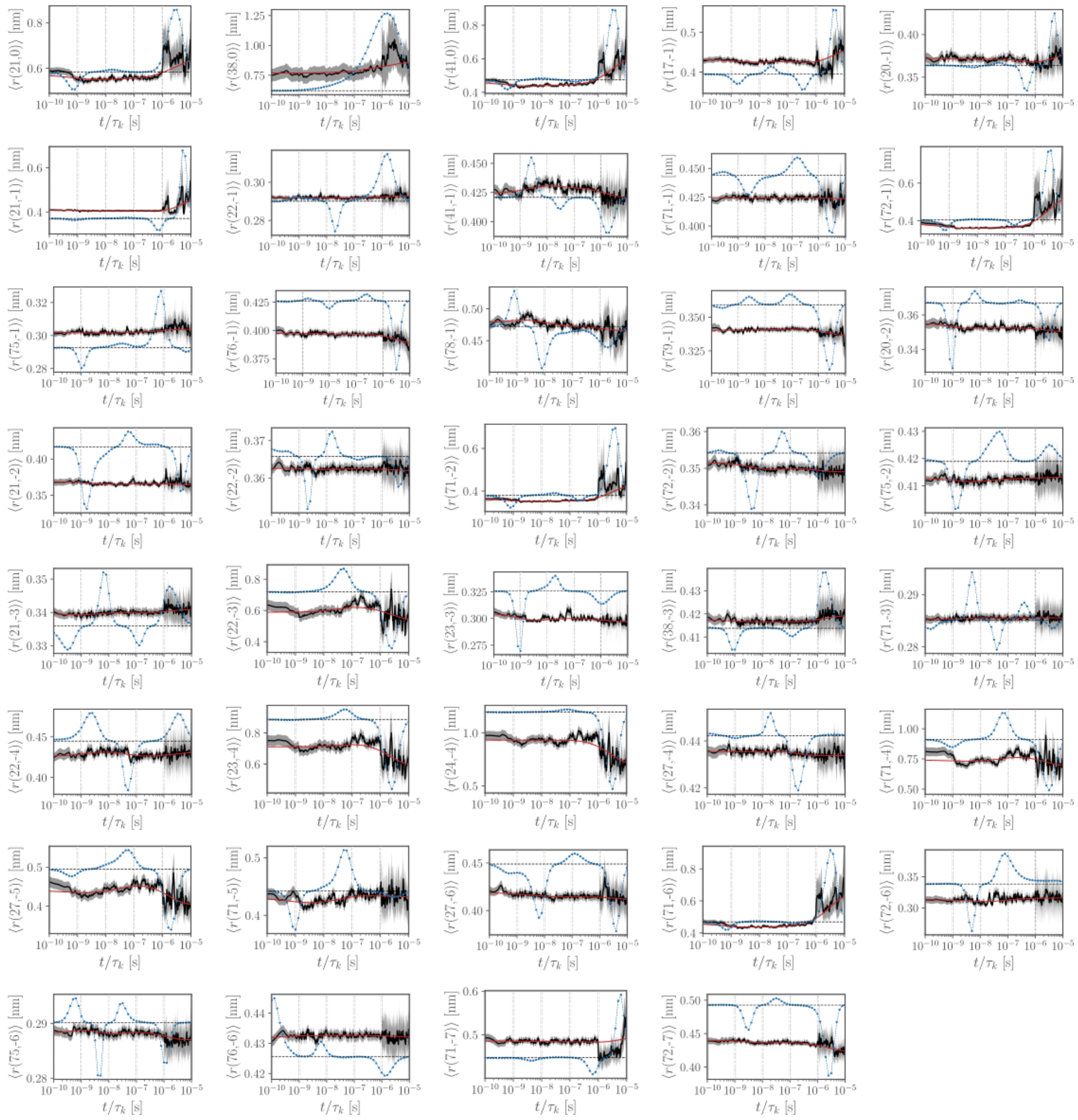
⟨r(21,0)⟩ [nm]
⟨r(38,0)⟩ [nm]
⟨r(41,0)⟩ [nm]
⟨r(17,-1)⟩ [nm]
⟨r(20,-1)⟩ [nm]
⟨r(21,-1)⟩ [nm]
⟨r(22,-1)⟩ [nm]
⟨r(41,-1)⟩ [nm]
⟨r(71,-1)⟩ [nm]
⟨r(72,-1)⟩ [nm]
⟨r(75,-1)⟩ [nm]
⟨r(76,-1)⟩ [nm]
⟨r(78,-1)⟩ [nm]
⟨r(79,-1)⟩ [nm]
⟨r(20,-2)⟩ [nm]
⟨r(21,-2)⟩ [nm]
⟨r(22,-2)⟩ [nm]
⟨r(71,-2)⟩ [nm]
⟨r(72,-2)⟩ [nm]
⟨r(75,-2)⟩ [nm]
⟨r(21,-3)⟩ [nm]
⟨r(22,-3)⟩ [nm]
⟨r(23,-3)⟩ [nm]
⟨r(38,-3)⟩ [nm]
⟨r(71,-3)⟩ [nm]
⟨r(22,-4)⟩ [nm]
⟨r(23,-4)⟩ [nm]
⟨r(24,-4)⟩ [nm]
⟨r(27,-4)⟩ [nm]
⟨r(71,-4)⟩ [nm]
⟨r(27,-5)⟩ [nm]
⟨r(71,-5)⟩ [nm]
⟨r(27,-6)⟩ [nm]
⟨r(71,-6)⟩ [nm]
⟨r(72,-6)⟩ [nm]
⟨r(75,-6)⟩ [nm]
⟨r(76,-6)⟩ [nm]
⟨r(71,-7)⟩ [nm]
⟨r(72,-7)⟩ [nm]
t/τ_k [s]

C2

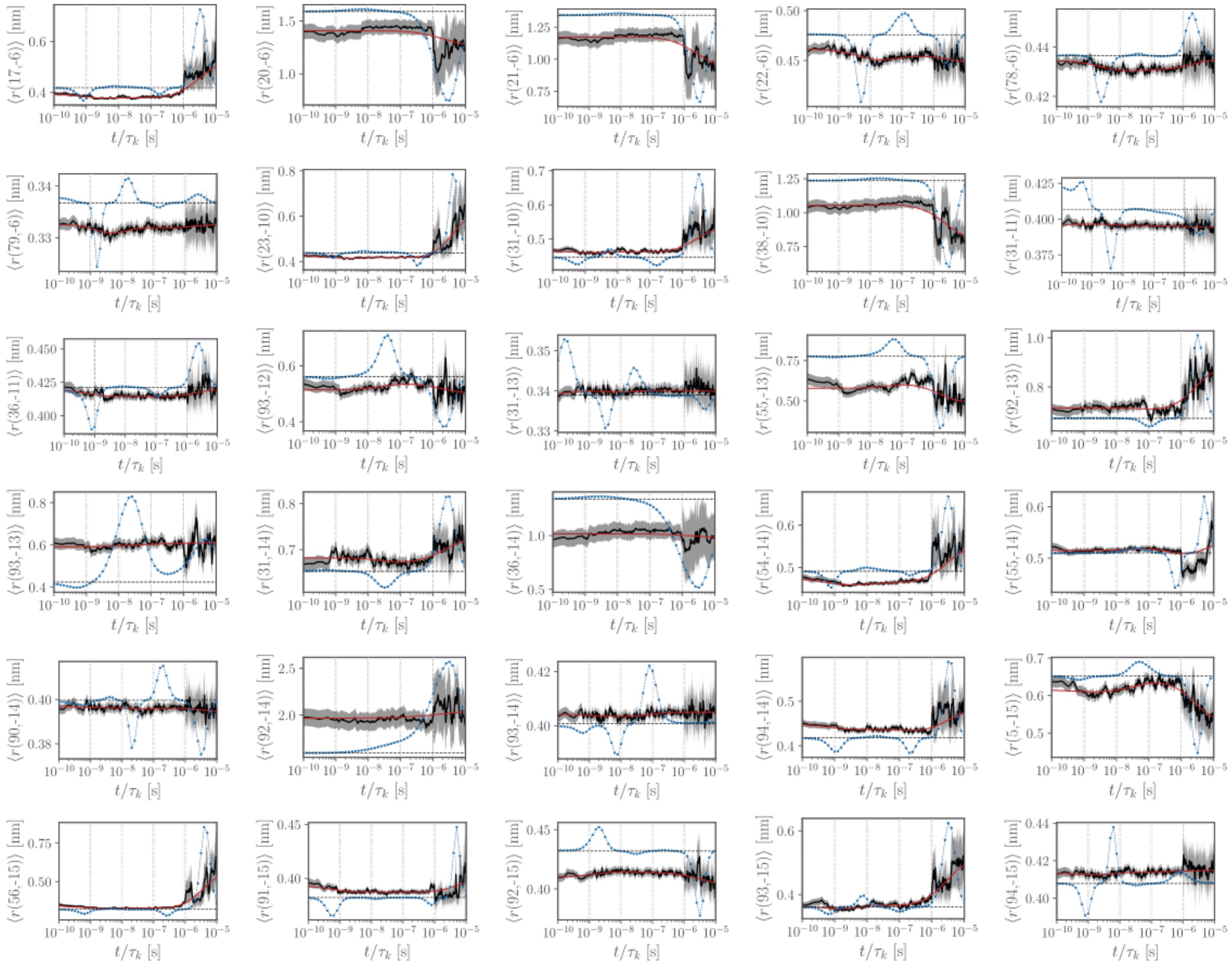
⟨r(17,-6)⟩ [nm]
⟨r(20,-6)⟩ [nm]
⟨r(21,-6)⟩ [nm]
⟨r(22,-6)⟩ [nm]
⟨r(78,-6)⟩ [nm]
⟨r(79,-6)⟩ [nm]
⟨r(23,-10)⟩ [nm]
⟨r(31,-10)⟩ [nm]
⟨r(38,-10)⟩ [nm]
⟨r(31,-11)⟩ [nm]
⟨r(36,-11)⟩ [nm]
⟨r(93,-12)⟩ [nm]
⟨r(31,-13)⟩ [nm]
⟨r(55,-13)⟩ [nm]
⟨r(92,-13)⟩ [nm]
⟨r(93,-13)⟩ [nm]
⟨r(31,-14)⟩ [nm]
⟨r(36,-14)⟩ [nm]
⟨r(54,-14)⟩ [nm]
⟨r(55,-14)⟩ [nm]
⟨r(90,-14)⟩ [nm]
⟨r(92,-14)⟩ [nm]
⟨r(93,-14)⟩ [nm]
⟨r(94,-14)⟩ [nm]
⟨r(5,-15)⟩ [nm]
⟨r(56,-15)⟩ [nm]
⟨r(91,-15)⟩ [nm]
⟨r(92,-15)⟩ [nm]
⟨r(93,-15)⟩ [nm]
⟨r(94,-15)⟩ [nm]
t/τk [s]

C3

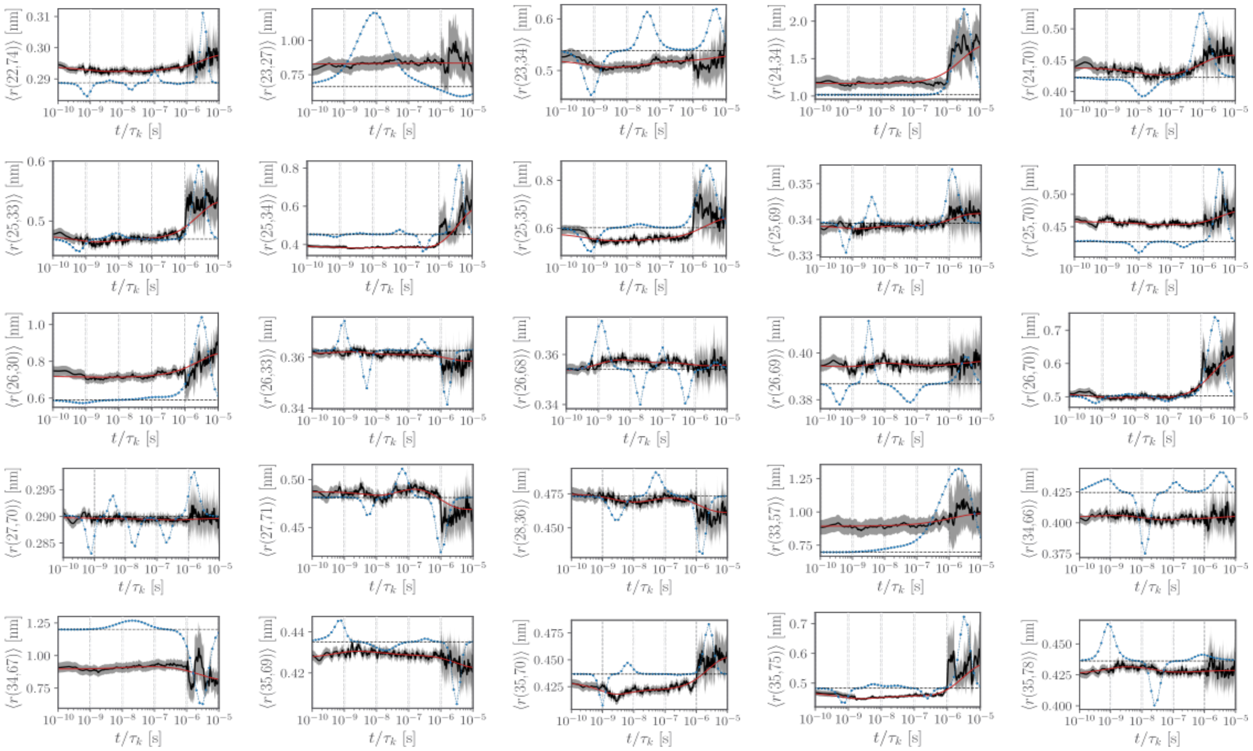

C4

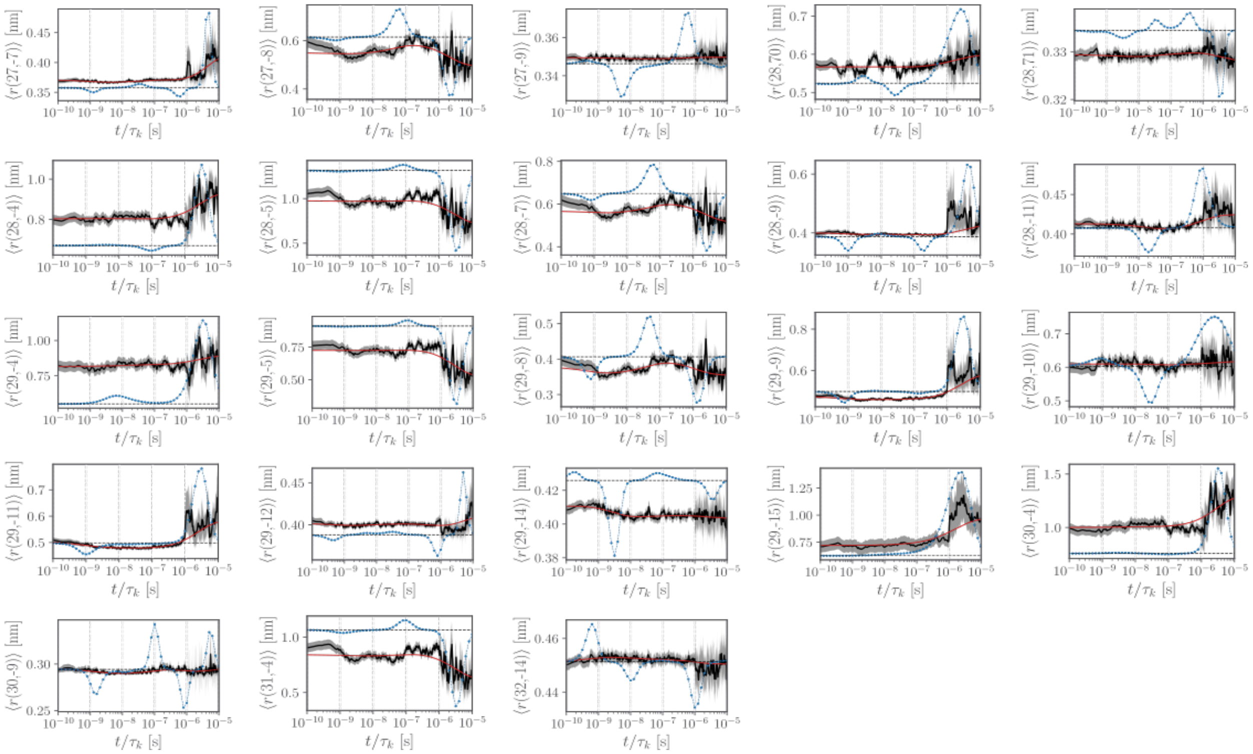

C5

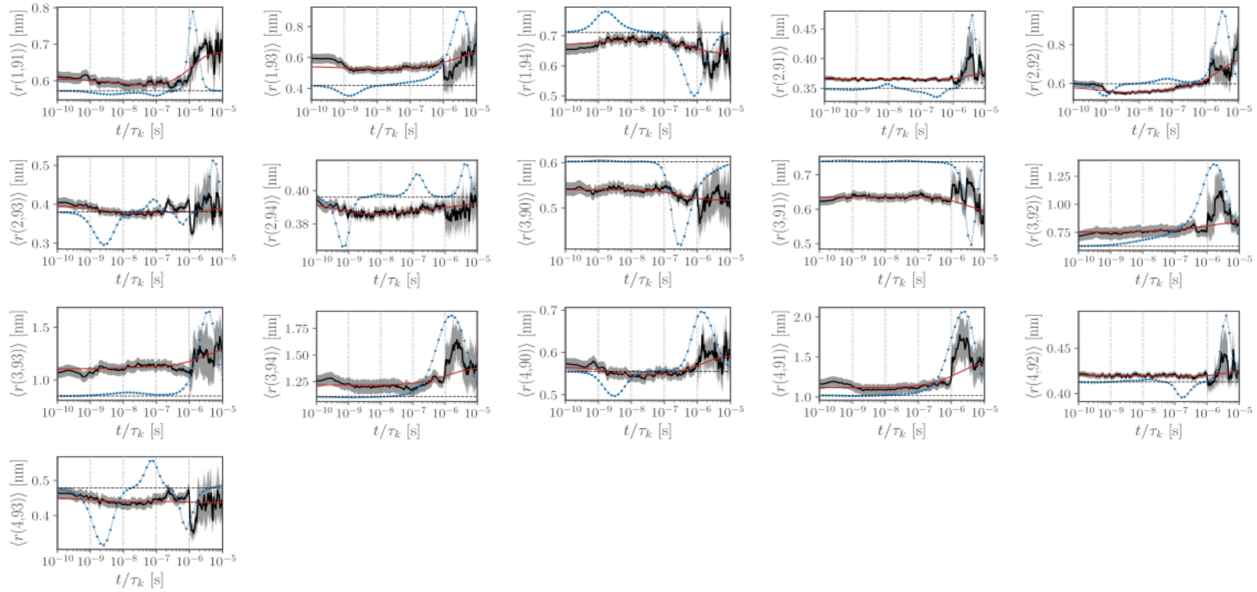

C6

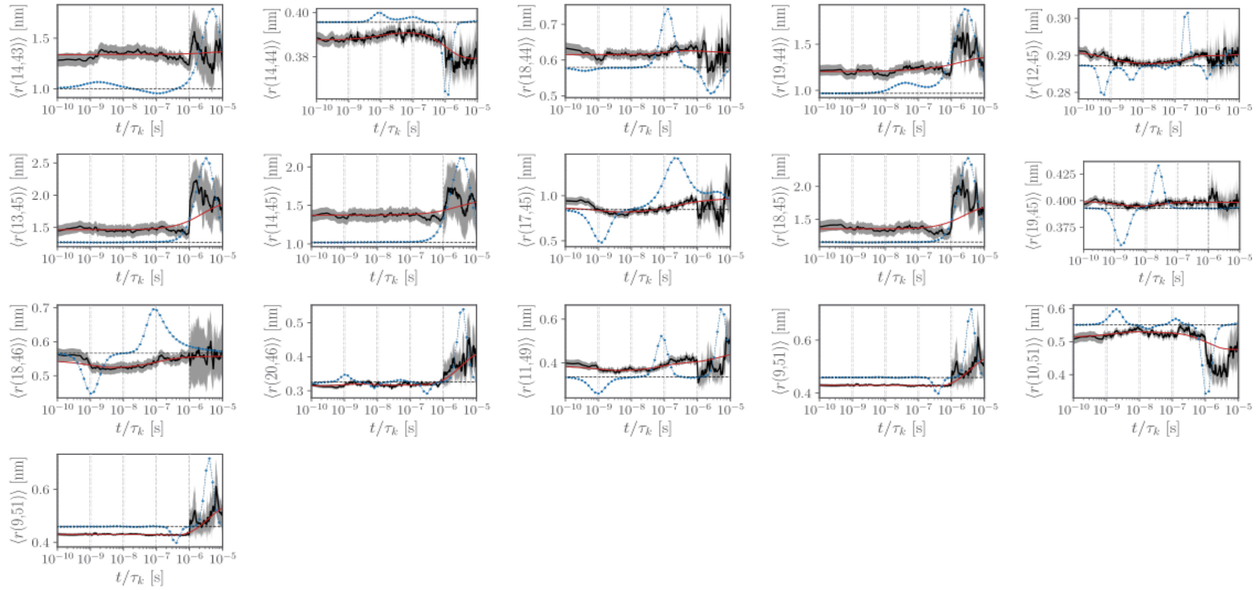

C7

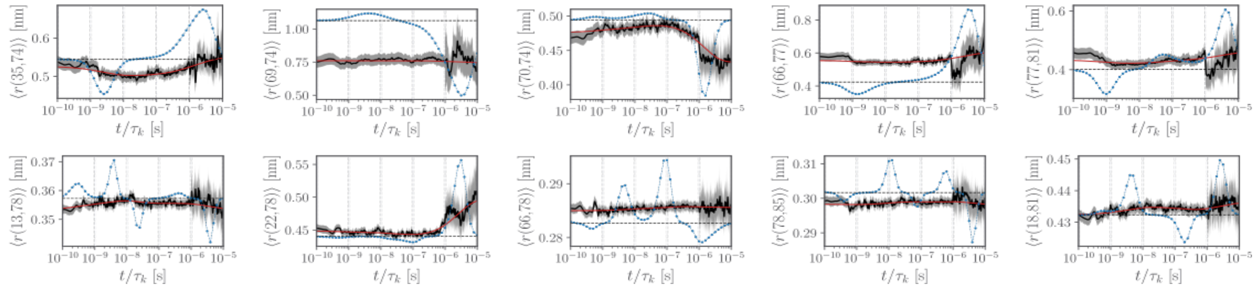

C8

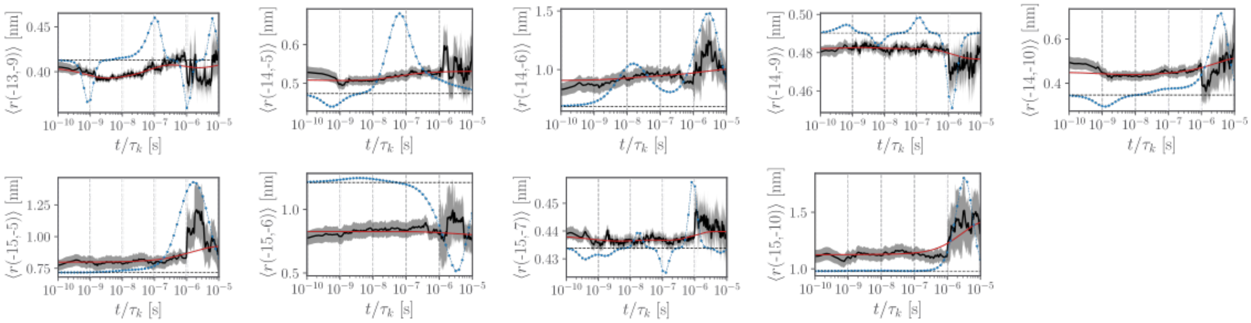

C9

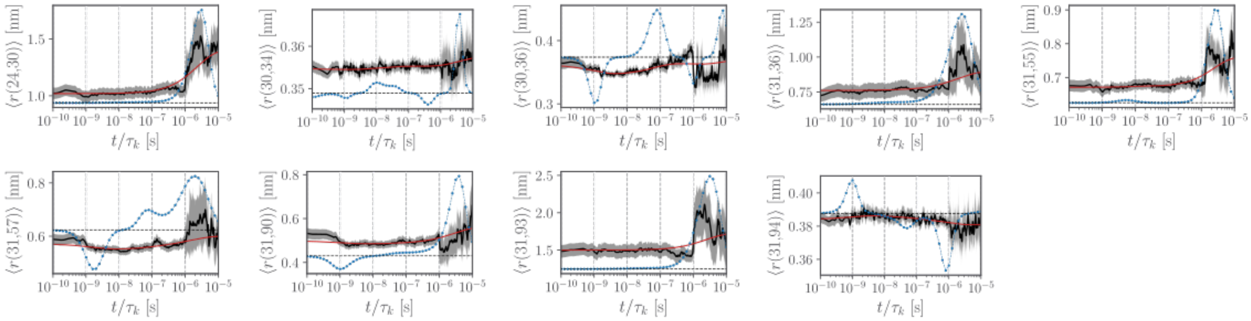

C10

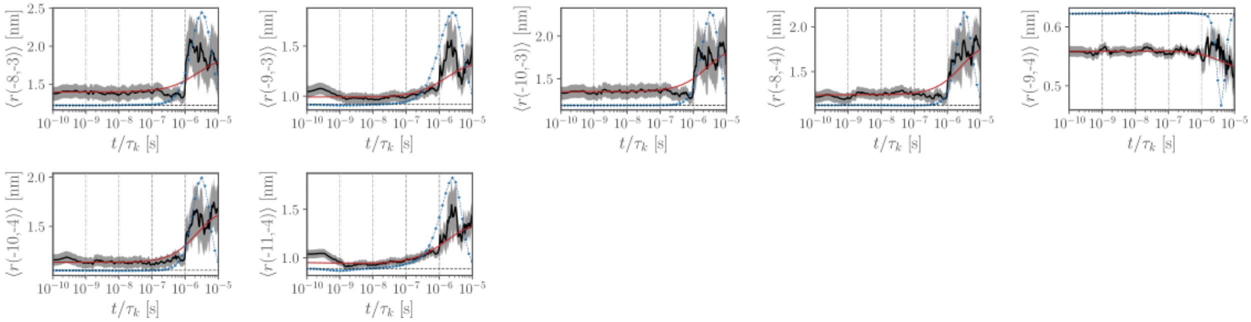

Figure S16: PDZ2L *cis*-to-*trans* – Timescale analysis of each contact distance with $\lambda = 100$ and 10 fit parameters per order of magnitude.

7 Diffusive dynamics

For all PDZ domains, we have found a characteristic slow dynamics on a timescale of a few microseconds. Following the idea that conformational transitions in proteins are largely governed by diffusive processes,[S12–S15] we fitted the microsecond dynamics of the contact distance $r_j(t)$ distances to a power law

$$r_j(t) = r_j^{(0)} + d_j\, t^{\alpha}.$$

Using three exemplary distances for each system, Fig. S17 shows that their long-time dynamics is indeed well captured by a power law. In all cases, distances from the two main clusters of each system (e.g., C1 and C6 for PDZ3) and a distance from cluster C3 were chosen. The exponent α is found to typically range between 0.3 (anomalous or subdiffusive motion) and 0.5 (normal diffusion), with the notable exception of distance $r(25, 72)$ of PDZ2S, for which a naive fit yields $\alpha = 1.34$. However, the rapid increase of this distance on a microsecond timescale is caused by a discrete structural rearrangement (i.e., the global opening of the binding pocket due to the rupturing the several contacts), and should not be confused with superdiffusive motion. In a similar way, cluster C1 and C6 of PDZ3 exhibit fast system-specific structural rearrangements, and are therefore only at long times fitted to a power law.

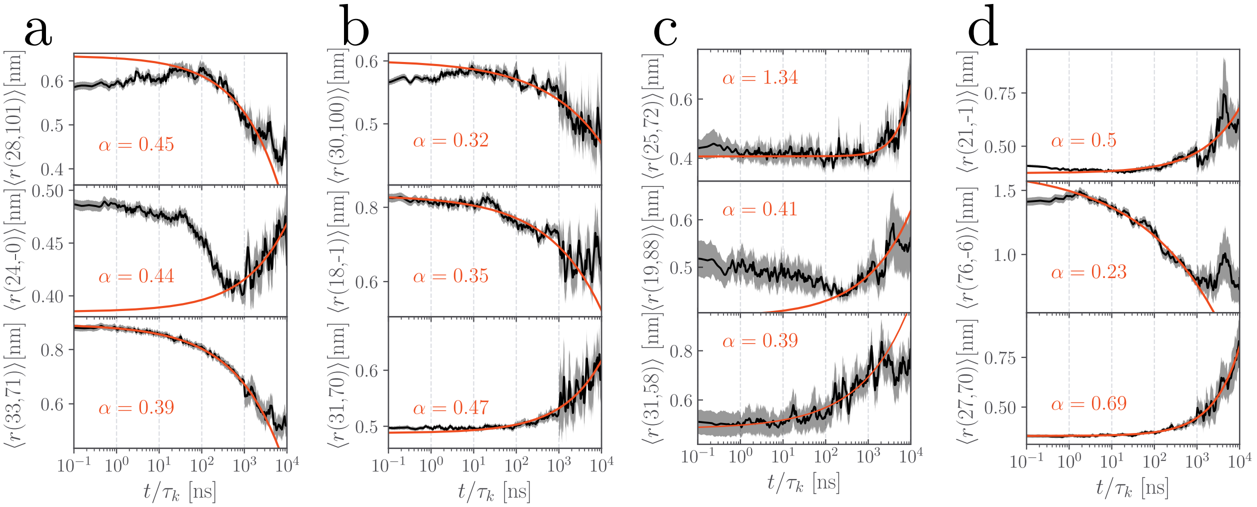

Figure S17: Time evolution of selected contact distances of the main clusters (black) fitted to a power-law for $t \geq 1\,\mu$s (red). Shown are results for (a) PDZ3, (b) PDZ3L6, (c) PDZ2S, and (d) PDZ2L.

References

(S1) Ali, A. A. A. I.; Gulzar, A.; Wolf, S.; Stock, G. Nonequilibrium Modeling of the Elementary Step in PDZ3 Allosteric Communication. *J. Phys. Chem. Lett.* **2022**, *13*, 9862–9868.

(S2) Ali, A. A. A. I.; Dorbath, E.; Stock, G. Allosteric communication mediated by protein contact clusters: A dynamical model. *J. Chem. Theory Comput.* **2024**, *20*, 10731 – 10739.

(S3) Nguyen, P. H.; Stock, G. Nonequilibrium molecular dynamics simulation of a photoswitchable peptide. *Chem. Phys.* **2006**, *323*, 36–44.

(S4) Diez, G.; Nagel, D.; Stock, G. Correlation-based feature selection to identify functional dynamics in proteins. *J. Chem. Theory Comput.* **2022**, *18*, 5079 – 5088.

(S5) Zhou, T.; Caflisch, A. Distribution of Reciprocal of Interatomic Distances: A Fast Structural Metric. *J. Chem. Theory Comput.* **2012**, *8*, 2930 – 2937.

(S6) Yang, L.; Song, G.; Jernigan, R. L. How Well Can We Understand Large-Scale Protein Motions Using Normal Modes of Elastic Network Models? *Biophysical Journal* **2007**, *93*, 920–929.

(S7) De Los Rios, P.; Cecconi, F.; Pretre, A.; Dietler, G.; Michielin, O.; Piazza, F.; Juanico, B. Functional Dynamics of PDZ Binding Domains: A Normal-Mode Analysis. *Biophys. J.* **2005**, *89*, 14 – 21.

(S8) Stevens, A. O.; He, Y. Allosterism in the PDZ Family. *Int. J. Mol. Sci.* **2022**, *23*, 1454.

(S9) Buchenberg, S.; Sittel, F.; Stock, G. Time-resolved observation of protein allosteric communication. *Proc. Natl. Acad. Sci. USA* **2017**, *114*, E6804–E6811.

(S10) Nagel, D.; Diez, G.; Stock, G. Accurate estimation of the normalized mutual information of multidimensional data. *J. Chem. Phys.* **2024**, *161*, 054108.

(S11) Bozovic, O.; Zanobini, C.; Gulzar, A.; Jankovic, B.; Buhrke, D.; Post, M.; Wolf, S.; Stock, G.; Hamm, P. Real-time observation of ligand-induced allosteric transitions in a PDZ domain. *Proc. Natl. Acad. Sci. USA* **2020**, *117*, 26031 – 26039.

(S12) Zwanzig, R. Diffusion in rough potentials. *Proc. Natl. Acad. Sci. (USA)* **1988**, *85*, 2029.

(S13) Neusius, T.; Daidone, I.; Sokolov, I. M.; Smith, J. C. Subdiffusion in Peptides Originates from the Fractal-Like Structure of Configuration Space. *Phys. Rev. Lett.* **2008**, *100*, 188103.

(S14) Best, R. B.; Hummer, G. Coordinate-dependent diffusion in protein folding. *Proc. Natl. Acad. Sci. USA* **2010**, *107*, 1088 – 1093.

(S15) Volkhardt, A.; Grubmüller, H. Estimating ruggedness of free-energy landscapes of small globular proteins from principal component analysis of molecular dynamics trajectories. *Phys. Rev. E* **2022**, *105*, 044404.
